# Midbrain tectal stem cells display diverse regenerative capacities in zebrafish

**DOI:** 10.1038/s41598-019-40734-z

**Published:** 2019-03-14

**Authors:** Benjamin W. Lindsey, Georgia E. Aitken, Jean K. Tang, Mitra Khabooshan, Alon M. Douek, Celia Vandestadt, Jan Kaslin

**Affiliations:** 1 Australian Regenerative Medicine Institute, Monash University Clayton Campus, Clayton, Victoria, 3800 Australia; 2 Present Address: Department of Human Anatomy and Cell Science, College of Medicine, University of Manitoba, Winnipeg, R3E 3P5 Canada

## Abstract

How diverse adult stem and progenitor populations regenerate tissue following damage to the brain is poorly understood. In highly regenerative vertebrates, such as zebrafish, radial-glia (RG) and neuro-epithelial-like (NE) stem/progenitor cells contribute to neuronal repair after injury. However, not all RG act as neural stem/progenitor cells during homeostasis in the zebrafish brain, questioning the role of quiescent RG (qRG) post-injury. To understand the function of qRG during regeneration, we performed a stab lesion in the adult midbrain tectum to target a population of homeostatic qRG, and investigated their proliferative behaviour, differentiation potential, and Wnt/β-catenin signalling. EdU-labelling showed a small number of proliferating qRG after injury (pRG) but that progeny are restricted to RG. However, injury promoted proliferation of NE progenitors in the internal tectal marginal zone (TMZi) resulting in amplified regenerative neurogenesis. Increased Wnt/β-catenin signalling was detected in TMZi after injury whereas homeostatic levels of Wnt/β-catenin signalling persisted in qRG/pRG. Attenuation of Wnt signalling suggested that the proliferative response post-injury was Wnt/β-catenin-independent. Our results demonstrate that qRG in the tectum have restricted capability in neuronal repair, highlighting that RG have diverse functions in the zebrafish brain. Furthermore, these findings suggest that endogenous stem cell compartments compensate lost tissue by amplifying homeostatic growth.

## Introduction

The adult stem cell niche is composed of heterogeneous neural stem and progenitor cells, reflecting their developmental origin, cell lineages, and proliferative dynamics^1–15^. Presently, the cellular and molecular signatures of these populations are best understood under homeostasis and repair within the vertebrate forebrain telencephalic niche^16–30^. Outside of the telencephalon we are only beginning to uncover the regenerative plasticity of stem/progenitor cells and their biological importance^31^, particularly in highly regenerative vertebrates^15^.

The zebrafish has emerged as a leading model of stem cell plasticity and regeneration with its wealth of neurogenic compartments positioned along brain ventricles^32–41^. Niches are populated by heterogeneous stem/progenitor phenotypes^16,17^, dominated largely by neuro-epithelial-like (NE) stem/progenitor cells and radial-glial cells residing in proliferative (pRG) or quiescent (qRG) states^15^. The striking regenerative ability of the zebrafish brain has given rise to the notion that most adult stem/progenitor cells are likely to be multipotent^15,20,38,39^, and as such, capable of replacing all cell lineages lost during injury (i.e. NE, RG, oligodendrocytes, neurons). While this hypothesis appears to be upheld by the largely quiescent Müller glia of the adult retina^42–44^, the unique regenerative profile of individual cell phenotypes across distinct stem cell niches of the brain is less clear.

Radial-glia of the dorsal telencephalon have been the focus of most injury studies in the zebrafish CNS^20,21,24,45–47^. We have shown that RG play a major role in regenerating new neurons that repopulate those lost, with these cells fated to become functional neuronal subtypes^20^. Interestingly, under homeostasis a large proportion of the dorsal RG population remain quiescent, regulated by high expression of Notch genes^22,48–50^. Downregulation of Notch signalling induces qRG to re-enter the cell cycle and increase symmetric division^48^, allowing them to respond to injury. In contrast, within the cerebellar niche RG are quiescent and do not serve as functional stem cells, with neurogenesis driven solely by multipotent NE-like stem cells under homeostasis^6^. We recently revealed that upon injury to the cerebellum, tissue regeneration was governed primarily by the NE population despite re-entry of qRG into the cell-cycle, recapitulating the homeostatic state of the niche^51^.

**Figure 1:**
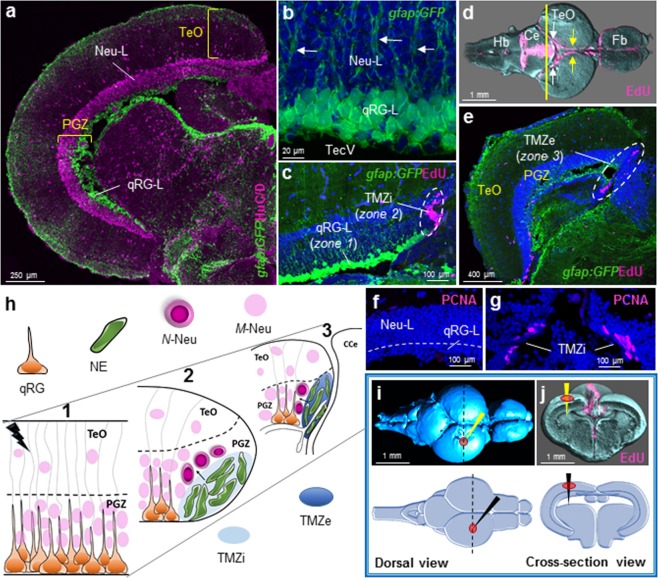
Tectal composition, stem cell niches, and stab lesion model. **(a)** Cross-sectional view of a single adult tectal hemisphere showing a laminar composition consisting of outer tectal superficial layers (TeO) and the deeper, cell-dense, periventricular grey zone (PGZ). The PGZ is subdivided into an upper neuronal layer (Neu-L, cytoplasmic HuC/D labelling; pink) and a quiescent radial-glial layer (qRG-L; *Tg(gfap:GFP)*^*mi2001*^ labelling; green) populating the roof of the tectal ventricle. **(b)** High magnification of the PGZ illustrating the 3–4 cell deep structure of the qRG-L (green) abutting the tectal ventricle (TecV) with radial processes extending upwards from qRG cells through the Neu-L (white arrows) and towards the superficial tectal layers. **(c)** Neuro-epithelial-like amplifying progenitor cells (NE; pink; zone 2) identified by EdU-labelling are located at the internal tectal marginal zone (TMZi; white dashed circle), adjacent to the qRG-L (green; zone 1). **(d)** Dorsal view of the homeostatic staining pattern of cell proliferation using EdU (pink) in reconstructed whole brains following Optical Projection Tomography (OPT). Image shows constitutively proliferating NE cells extending the length of the tectum from the TMZi where amplifying NE progenitor cells reside (yellow arrows), to the external tectal marginal zone (TMZe; white arrows) where more slowly proliferating NE cells have been identified^48,49^. Hb, hindbrain; Ce, cerebellum; TeO, optic tectum; Fb, forebrain. Yellow line depicts cross-sectional level shown in panel **(e)**. **(e)** Cross-section view of the TMZe (white dashed circle; zone 3) labelled with EdU (pink), where populations of slow cycling NE exist at the posterior aspect of the adult midbrain adjacent to the medially located cerebellum. **(f**,**g)** Proliferating Cell Nuclear Antigen (PCNA) immunolabelling confirming the same constitutive pattern of cell division seen with EdU (compare with panel **c**) in the qRG-L and TMZi. **(h)** Schematic representing the three stem cell zones (1–3) investigated following tectal stab lesion (lightning bolt) superficial to the underlying qRG layer: 1 – qRG population (orange) lining the tectal ventricle; 2 – proliferating NE amplifying progenitors (green) located at the TMZi (light blue) adjacent the qRG population; 3 – slowly cycling NE cells located in the caudal TMZe (dark blue). Note that NE cells of zones 2–3 proliferate under homeostatic conditions, with zone 2 NE amplifying progenitors producing lifelong newborn neurons (*N*-Neu). *M*-Neu, mature neuron; CCe, corpus cerebelli. **(i,j)** Dorsal (**i**) and cross-sectional (**j**) views of the intact control brain depicting the neuroanatomical level (**i**, black dashed line) and cannula insertion site (red circles; black and yellow arrows) for our tectal stab lesion into the left hemisphere in OPT reconstructed brains (top) and schematic images (bottom). In panels b,c,e–g, DAPI nuclear counterstaining (blue) was performed. In panels c–g, EdU was pulsed twice over a 4-hr chase period prior to sacrifice. In all cross-sectional and whole brain images dorsal is oriented up.

Distinct from other major structures of the adult CNS, the zebrafish midbrain tectum contains stem cell niches populated entirely by a single stem/progenitor cell type^34^. Here, an extensive population of qRG exist at the roof of the tectal ventricle, while NE amplifying progenitors that contribute to lifelong neurogenesis are found at the internal tectal marginal zone (TMZi)^34,52^. Embryonically these cells are derived from slow-amplifying progenitors^52,53^. Recently it has been demonstrated that NE amplifying progenitors of the TMZi are the last of a well-defined NE lineage that originate from *Her5-*positive NE stem cells in the external tectal marginal zone (TMZe) of the posterior tectum^54^. Moreover, evidence suggests that NE cells of the zebrafish tectum rely on Wnt/β-catenin signalling to promote stem cell proliferation towards a neuronal phenotype^55^, similar to the role of Wnt in adult mammalian stem cell niches^56–58^. At present, the regenerative capacity of qRG and NE cells within the midbrain tectum (see Fig. 1c,e), along with the signalling pathways governing their regulation, remain largely unknown.

In this study, we designed a tectal stab lesion assay to examine the proliferative dynamics, neurogenic potential, and requirement of Wnt/β-catenin signalling during the regenerative response in three distinct stem cell zones (see Fig. 1h): the quiescent radial-glial layer containing non-proliferating qRG (*zone 1*), the TMZi containing NE amplifying progenitors (*zone 2*), and the TMZe housing more slowly cycling NE progenitors (*zone 3*). We show that while a modest population of normally quiescent ventricular RG can be stimulated to re-enter the cell cycle and produce EdU^+^ progenitors, these progenitors are restricted to producing RG. No new neurons were detected at the lesion site after injury. However, mechanical injury was sufficient to promote upregulation of constitutive levels of proliferation and neurogenesis from NE amplifying progenitors at the TMZi, implicating this population in neuroregeneration as previously demonstrated in the cerebellum^51^. Nuclear β-catenin staining, along with the downstream target gene of Wnt/β-catenin signalling, *axin2*, remained unchanged between uninjured and injured states in the qRG layer of the midbrain, but were elevated in the TMZi. Following tectal lesion, attenuation of Wnt/β-catenin signalling via its antagonist, *Dickkopf-1*, revealed no change in the proliferative response of EdU^+^ cells in the quiescent RG layer of the PGZ, nor the NE population at the TMZi, suggesting a Wnt-independent proliferative response. Taken together, our results contribute to the notion that the regenerative potential of the adult zebrafish CNS is orchestrated by discrete stem/progenitor phenotypes that are regulated by intrinsically-defined regenerative programs.

## Results

### The adult optic tectum contains an isolated population of non-cycling quiescent radial-glial cells lining the roof of the tectal ventricle

Quiescent radial-glial (qRG) stem/progenitor cells comprise a proportion of many heterogeneous adult stem cell niches throughout the teleost brain^6,11,15^. Within the optic tectum, an extensive, isolated population of qRG cells are uniquely positioned adjacent to the tectal ventricle^34^. These constitute the quiescent radial-glial layer (qRG-L) below the upper neuronal layer (Neu-L) of the periventricular grey zone (PGZ; Fig. 1a). Reporter lines for glial fibrillary acidic protein (GFAP; Tg(gfap:GFP)^*mi2001*^ Zebrafish Information Resource Centre; ZIRC) and *her4*.*3*^59^ (Tg(*her4*.*3*:EGFP)^y83^; previously known as *her4*.*1*), as well as antibodies against glial markers glutamine synthetase (GS), S100β, and fabp7a^34^, show that the qRG-L forms a 2–3 cell deep ruffled epithelium. Individual cell processes of these qRG project upwards through the dense neuronal layer of the PGZ (Fig. 1b, white arrows), towards the superficial tectal layer^60,61^. Injections of the proliferative marker, 5-ethynyl-2′-deoxyuridine (EdU), that is incorporated during the *S*-phase of the cell cycle when active DNA synthesis takes place, demonstrate that unlike actively proliferating RG cells (pRG) of the telencephalon, glia of this layer do not exist within a proliferative state^30^ (Fig. 1c; qRG-L – zone 1). EdU, however, is incorporated by neuro-epithelial-like (NE) amplifying progenitor cells located at the internal tectal marginal zone (TMZi) which contribute to constitutive neurogenesis throughout life^34^ (Fig. 1c; TMZi – zone 2). Similar findings have previously been shown in the optic tectum of the medaka^62^. Whole brain EdU-staining shows that NE progenitors are situated along the length of the adult TMZ, forming a ribbon of cells at the medial edge of each hemisphere that begins at the TMZi (Fig. 1d, yellow arrows) and continues posteriorly to the external tectal marginal zone (TMZe), adjacent to the cerebellum (Fig. 1d, white arrows). Cross-sections through the posterior tectum (yellow line) reveal that a large pool of EdU^+^ NE progenitor cells are detected in the TMZe (Fig. 1e; dashed circle), from which the tectal NE lineage and RG cells are derived^54^. NE progenitor cells in the TMZe have been shown to be slowly cycling in nature compared with those of the TMZi^52,53^. Antibody labelling using Proliferating Cell Nuclear Antigen (PCNA), a cell cycle marker expressed from late G_1_ phase to M-phase^63,64^, further confirm the absence of cycling cells in the qRG layer (Fig. 1f; qRG-L), and constitutive rates of NE proliferation in the TMZi (Fig. 1g). These distinctive stem/progenitor niches within the adult optic tectum provide an exceptional experimental system to investigate how each population (Fig. 1h; 1 *– qRG; 2 – NE amplifying progenitors; 3 – NE slowly cycling progenitors;* see also Fig. 1c,e for location of zones) responds to injury and contributes to regenerative neurogenesis.

To address this question, we designed a tectal stab lesion assay whereby a cannula (i.e. 30 G syringe needle), positioned dorsomedially in a single tectal hemisphere, was inserted vertically into the neurocranium through the tectal layers, terminating at the ventricle (Fig. 1I,j; top: OPT-rendered brain; bottom: schematic view of lesion coordinates). To reach the qRG population at the tectal ventricle it was necessary to penetrate through the dense neuronal layer of the upper PGZ. The use of syringe needles of this diameter or larger to perform stab injuries in the adult zebrafish brain has been well established and shown to successfully elicit a proliferative response from resident stem cell populations^20,21,24^. We reasoned that the location of our injury site was optimal to elicit a response from the underlying qRG cells in the PGZ, and was in close enough proximity to the NE amplifying (NE-Ap) population for these cells to respond to injury-induced signals. Moreover, the posterior directional flow of cerebrospinal fluid through the tectal ventricle provides a direct path of transmission of signals arising from the lesion site to contact NE cells adjacent to the ventricle in the TMZe.

To characterize our tectal stab lesion assay, we took advantage of our recently developed whole brain EdU staining technique that allows visualization of dividing cells in the optically transparent brain following Optical Projection Tomography (OPT) scanning and reconstruction^40,65^. Using IMARIS imaging software, we designed an algorithm facilitating quantification of total EdU volume in consecutively larger spherical rings emanating from the site of injury in the tectum (i.e. centre-point; see Supplementary Fig. S1a–i and *Optical Projection Tomography (OPT) Imaging and Analysis* in the methods section for further details). By calculating the proportion of EdU volume/sphere (6 spheres total) at early time points post-lesion, including 1-dpl and 3-dpl, we were able to extract patterns in the global proliferative response of the tectal hemisphere. Three-dimensional sphere analysis of the distribution of EdU^+^ staining radiating from the lesion site over the first 3-days post-injury (**see** Supplementary Fig. S1j**;** control, *n = *6 brains; 1-dpl, *n = *8 brains; 3-dpl, *n = *8 brains) revealed a progressive decline in the proportion of EdU volume/sphere at 1-dpl from ~ 55% to 25%, with a significant difference between sphere distance 0–49 µm and 250–299 µm (see Supplementary Fig. S1k,l). By contrast, we detected a significant reduction in the proportion of EdU volume/sphere between the sphere nearest the lesion site (i.e. 0–49 µm sphere) and those more distal at 3-dpl (see Supplementary Fig. S1m,n), suggesting that injury-induced cell proliferation was most pronounced nearest the lesion site at all time points. Comparison across time points further illustrates that the proliferative response to tectal injury is markedly higher distal to the lesion site at 1-dpl compared to 3-dpl (see Supplementary Fig. S1k and S1m).

To assess how widespread the proliferative response was throughout the midbrain following injury to a single tectal hemisphere, we additionally examined PCNA antibody labelling at 3-dpl (see Supplementary Fig. S2a; control, *n* = 3 brains; 3-dpl, *n* = 3 brains). Compared to control animals where modest PCNA labelling was seen only in the valvula cerebelli of the cerebellum (white arrows; see Supplementary Fig. S2b), cross-sections at 3-dpl at the level of the lesion illustrated robust increases in cell proliferation in domains of the dorsal and ventral midbrain of both hemispheres (see Supplementary Fig. 2c). In the lesioned hemisphere, PCNA^+^ cells were most pronounced at the site of injury of the tectum (yellow asterisk; white dashed circle), within the valvula cerebelli (white arrows), at midline domains (white square), and in particular, markedly upregulated in the hypothalamus (white bracket). With the exception of tectal proliferation and fewer PCNA^+^ hypothalamic cells (red bracket), the unlesioned hemisphere demonstrated proliferation in similar domains as the injured hemisphere. To more directly examine the anterior-posterior proliferative response post-injury within the PGZ, where the quiescent RG layer resides, we sectioned lesioned midbrains ~ 50 µm anterior and posterior to the lesion site and documented levels of PCNA labelling (see Supplementary Fig. 2d–f’). PCNA^+^ staining was greatest directly below the site of lesion in the quiescent RG layer, however conspicuous levels of cell proliferation were readily visible both anterior and posterior to the site of lesion in this same domain. These data demonstrate that signals arising from the lesion site produce a far-reaching proliferative response at or surrounding the quiescent RG layer of the injured tectal hemisphere.

The above findings led us to ask whether the global proliferative response of the brain to injury might be conserved independent of the lesion site. We recently showed that forebrain telencephalic stab injury leads to a temporal pattern of brain-wide cell proliferation that is globally upregulated at 1-dpl, but becomes restricted to the injury site by 3-dpl^40^. To this end, by comparing our telencephalic^20^ and tectal stab lesion models following OPT whole brain staining and EdU imaging^40,65^, we consistently observed upregulation of EdU at 1-dpl across the neuro-axis before becoming restricted to the lesion site at 3-dpl, and resembling uninjured (i.e. control) levels by 7-dpl (see Supplementary Fig. S3a,b). This result may imply that the vertebrate brain activates an acute, brain-wide stereotypical proliferative response to injury, prior to acting specifically at the site of injury.

**Figure 2:**
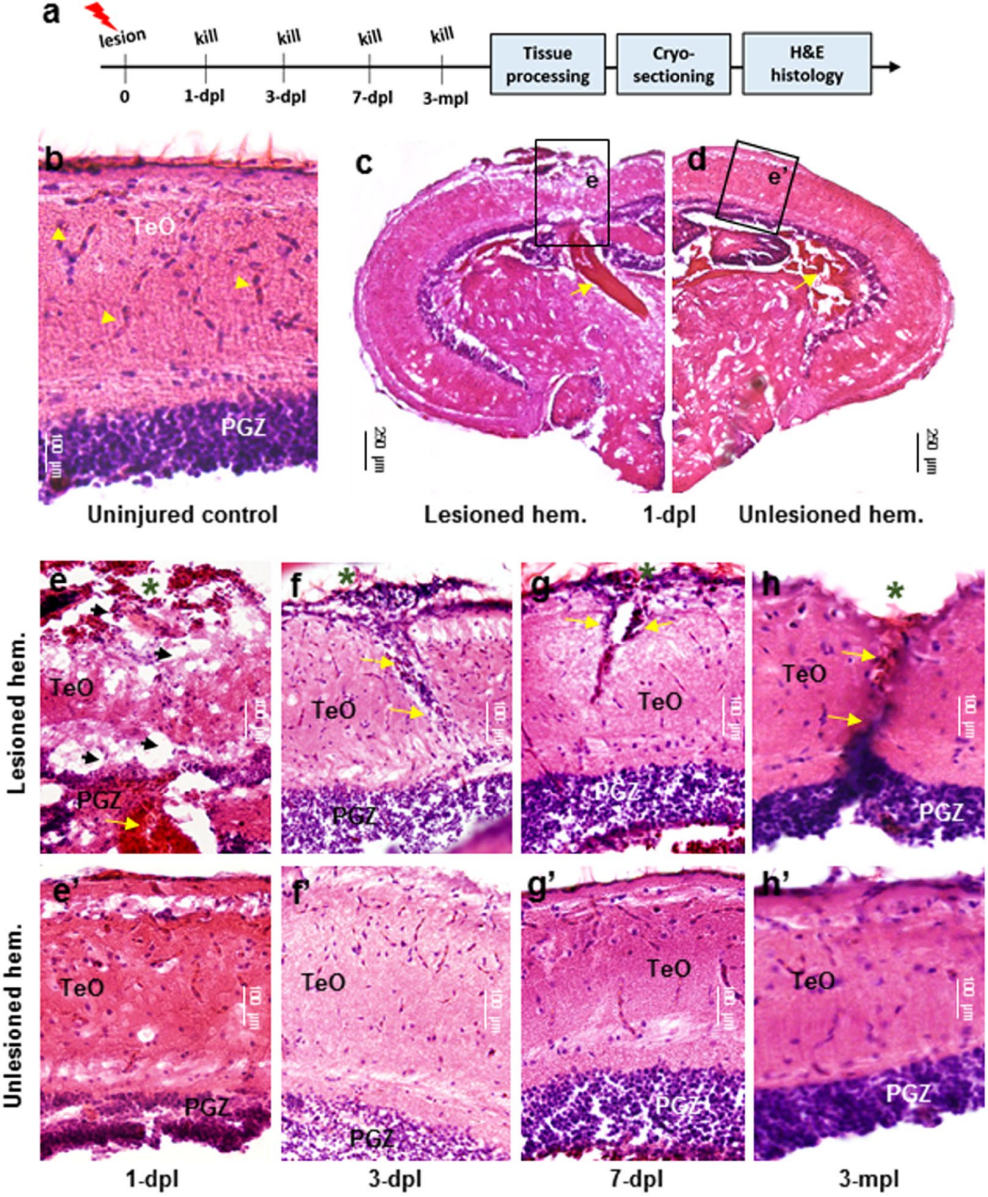
Histological response following tectal lesion. **(a)** Experimental design for hematoxylin and eosin (H&E) staining following lesion. **(b)** Control tectum showing well organized PGZ and highly vascularized superficial TeO layers (yellow arrowheads). **(c**,**d)** Overview of the tectal response to stab lesion in the lesioned **(c)** and unlesioned **(d)** hemispheres at 1-dpl. At low magnification most conspicuous is the blood pooling immediately below the lesion site and the highly disrupted TeO and PGZ compared to the unlesioned hemisphere. Higher magnification views are demarcated by black boxes for lesioned **(e)** and unlesioned (e’) hemispheres. (**e-**e’) Tissue 1-dpl in the lesioned hemisphere **(e)** showed extensive damage across tectal layers (TeO + PGZ) following cannula insertion, the presence of vacuoles at the lesion site (black arrowheads), and extensive blood pooling underlying the fragmented PGZ (yellow arrows). Despite a slight disruption in the PGZ, the unlesioned hemisphere (e’) closely resembles uninjured control tissue **(b)**. (**f**-f’) Tissue at 3-dpl in the lesioned hemisphere **(f)** was characterized by a reduction in the size of vacuoles and oedema, along with a pronounced increase in the number of cell bodies within the lesion canal (yellow arrows). However, already at 3-dpl the PGZ begins to resembled the unlesioned hemisphere. The unlesioned hemisphere (f’) appeared similar to 1-dpl (e’). (**g-**g’) Tissue at 7-dpl in the lesioned hemisphere **(g)** demonstrated a reduction in the number of cell bodies from within the lesioned canal, but showed that both sides of the canal have yet to re-joined together (yellow arrows). No change in the unlesioned hemisphere from 3-dpl was noted (g’). (**h**-h’) While complete tissue recovery was not achieved by 3-mpl in the lesioned hemisphere **(h)**, tissue had re-joined together and both the superficial TeO layers and PGZ closely resembled control conditions. At 3-mpl, the unlesioned hemisphere was indistinguishable from the uninjured control **(b)**. Cross-sectional views with dorsal oriented up are shown for all histological images. In (**e–h**), green asterisks denote the site of lesion. dpl, days post lesion; mpl, months post lesion.

To determine the histological response of the tectum to injury and the time course of repair, we stained sections with hematoxylin and eosin (H&E) at early (first week) and late (3-months) time points to assess recovery (Fig. 2a; control, *n* = 3; 1-dpl, *n* = 5; 3-dpl, *n* = 5; 7-dpl, *n* = 4; 3-mth, *n* = 3). Compared to control tissue that demonstrated well delineated tectal layers (TeO; PGZ) and vasculature (yellow arrowheads; Fig. 2b), at 1-dpl tectal injury led to marked disorganization of the superficial tectal layers and underlying PGZ in the lesioned hemisphere (Fig. 2c). In the unlesioned hemisphere (Fig. 2d), slight disruption in the organization of the PGZ was also observed compared with control tissue (*see* Fig. 2b). Blood pooling below the lesion site, as well as within the tectal ventricle of the unlesioned hemisphere was further detected (Fig. 2c,d; yellow arrows). At higher magnification, the injured hemisphere appeared highly disrupted immediately at the lesion site with extensive bleeding leading to the presence of oedema extending ventrally from the tectal ventricle into the underlying parenchyma (Fig. 2e; yellow arrows) compared to the unlesioned hemisphere (Fig. 2e’). Compared with control tissue (*see* Fig. 2b) and the unlesioned hemisphere at 1-dpl (Fig. 2e’), we further documented an increase in the number of vacuoles (black arrowheads) and a loss of vascularization in the damaged tissue (Fig. 2e). By 3-dpl, a conspicuous stab canal at the site of lesion was present (Fig. 2f) but otherwise tissue adjacent the injury began to resemble that of the unlesioned hemisphere (Fig. 2f’), including the PGZ. Blood clotting was no longer observed, damaged tissue was less vacuolated, and the vertical lesion formed by the cannula filled with a conspicuous number of cell bodies (Fig. 2f; yellow arrows). Histological analysis of the lesioned hemisphere at 7-dpl displayed a reduction in the size of the lesion canal along with a notable decrease in eosin-positive cell bodies (Fig. 2g). With the exception of the lesion canal, and slightly disrupted PGZ layer, tissue organization was for the most part similar to the unlesioned hemisphere (Fig. 2g’) and uninjured control (*see* Fig. 2b). By 3-months post-injury, tissue from both sides of the lesion canal had re-joined together (Fig. 2h; yellow arrows), but had yet to fully recover to resemble the unlesioned hemisphere (Fig. 2h’). However, at this time, the PGZ of both the lesioned and unlesioned hemispheres were indistinguishable in their density of eosin-positive cell bodies compared with control tissue (*see* Fig. 2b).

The infiltration of cell bodies into the lesion canal at 3-dpl observed during histological analysis prompted us to ask whether the extensive number of cells within the lesion site might be a result of the rapid immune response known to occur following CNS injury. Indeed, examination of the tectal injury site using the *Tg(mpeg1:mCherry)*^*g122*^ transgenic reporter line that labels brain microglia and peripheral macrophages^66^ (see Supplementary Fig. S4a; control, *n* = 3; 1-dpl, *n* = 4; 3-dpl, *n* = 4), showed that unlike control conditions where few resident microglia were present (see Supplementary Fig. S4b), at 1-dpl a large number of macrophages were recruited to the lesion site and lined the vertical lesion canal (see Supplementary Fig. S4c). A large population of macrophages were also observed below the lesion site in the PGZ layer. At 3-dpl the majority of these cells were located within the canal and PGZ (see Supplementary Fig. S4d), accounting, at least in part, for the increase in cell bodies observed from histological analysis at this same time point. Along with recruitment of macrophages towards the lesion canal following injury we also noted a change in the cellular morphology of macrophages from elongated cell bodies with ramified processes (see Supplementary Fig. S4b**;** red circle) to a condensed, amoeboid-like morphology (see Supplementary Fig. S4c,d, red circles**;** also see higher magnifications in Supplementary Fig. S4e–g, red arrows). This morphological change is indicative of a shift from a surveillance role under homeostasis to activation of these macrophages post-lesion^67^. EdU labelling demonstrated that compared with unlesioned tissue, where all microglia remain in a non-proliferative state (see Supplementary Fig. S4b,e; white box and red arrows), a fraction of macrophages (microglia or infiltrated peripheral macrophages) double-labelled with EdU at 1-dpl and 3-dpl following short 4-hour chase periods (see Supplementary Fig. S4c,d,f,g; white boxes and red arrows). Proliferating macrophages were detected primarily in the superficial layers of the tectum. High magnification images show both the condensed morphology of these cells and their mCherry^+^/EdU^+^ proliferative status (see Supplementary Fig. S4f,g; red arrows). These findings were further confirmed by repeating this experiment using the pan-leukocyte marker, L-plastin^68^, in combination with the proliferative marker PCNA. By comparing the unlesioned (see Supplementary Fig. S4h) and lesioned (see Supplementary Fig. S4i) hemispheres at 3-dpl, we detected clear co-labelling of these markers in a subpopulation of resident macrophages (see red arrows in Supplementary Fig. S4i) that reflected the same pattern observed using the *mpeg1:mCherry* reporter line.

### Injury stimulates a subpopulation of quiescent radial-glial cells to re-enter the cell cycle and upregulates proliferation from neuro-epithelial-like progenitors of the TMZi

**Figure 3:**
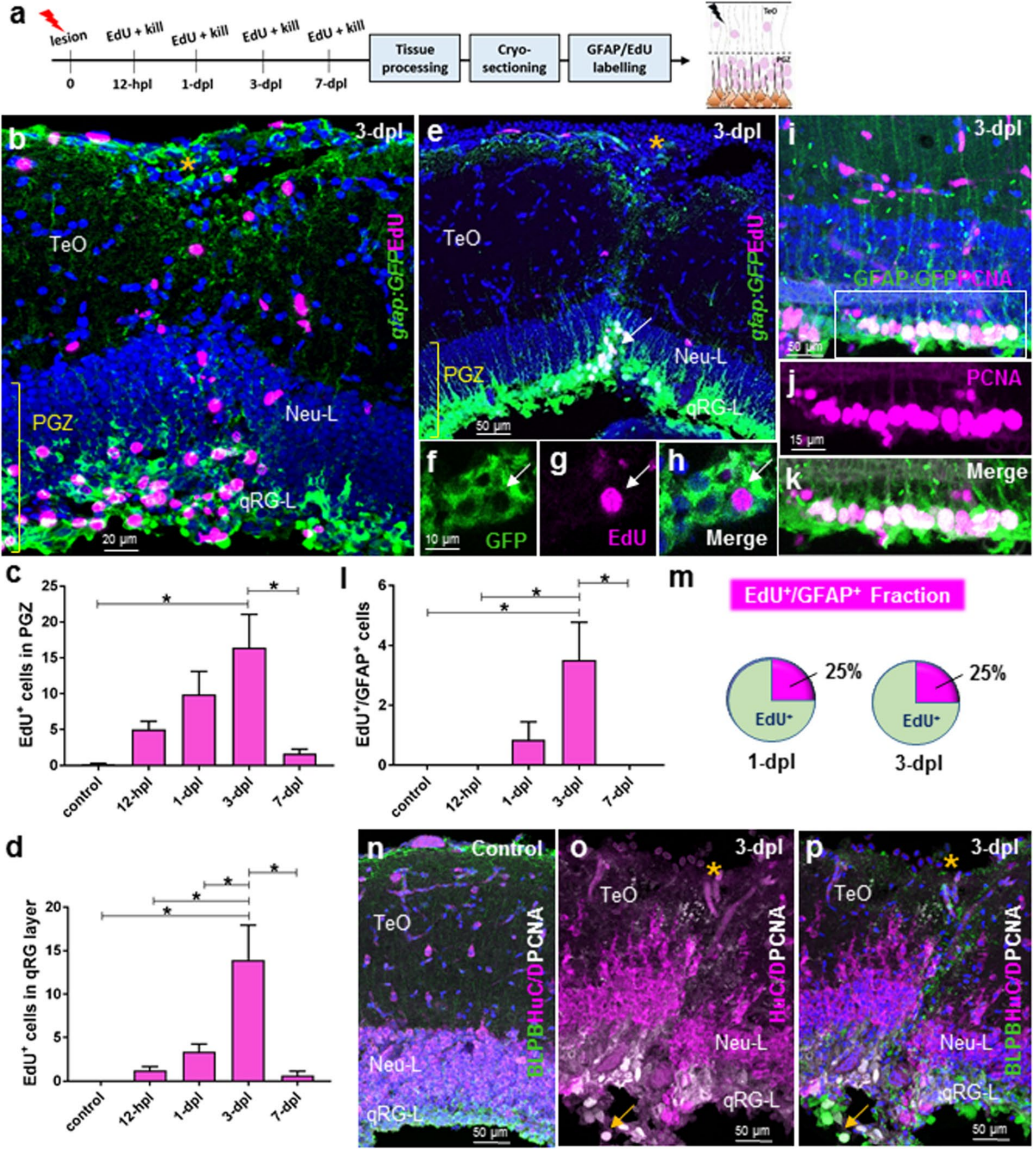
Cell proliferation post-lesion in the qRG layer of the periventricular grey zone (PGZ; stem cell zone 1). **(a)** Experimental design to investigate EdU proliferation arising from the qRG populations. **(b)** Example of the proliferative response at the lesion site (orange asterisk) in the PGZ (high proliferation) and superficial layers (modest proliferation) at 3-dpl when response is maximal. **(c)** Total EdU^+^ cells in the PGZ (Neu-L + qRG-L) at times post-lesion. One-way ANOVA; F (4, 23) = 4.395, *p* = 0.0087; Tukey’s multiple comparisons test: 3-dpl vs control, *p* = 0.0109; 3-dpl vs 7-dpl, *p* = 0.0365. **(d)** Total EdU^+^ cells in the qRG layer at times post-lesion. One-way ANOVA; F (4, 24) = 8.585, *p* = 0.0002; Tukey’s multiple comparisons test: 3-dpl vs control, *p* = 0.0005; 3-dpl vs 12-hpl, *p* = 0.0008; 3-dpl vs. 1-dpl, *p* = 0.0031; 3-dpl vs 7-dpl, *p* = 0.0018. **(e)** Example of a population of co-labelled *gfap:GFP*^+^*/*EdU^+^ cells in the qRG layer of the PGZ at 3-dpl (qRG-L, white arrow). High magnification view of proliferating radial-glia (pRG) in separate GFP (**f**) and EdU (**g**) channels, and merge (**h**) at 3-dpl. **(i–k)** Co-labelled population of *gfap:GFP*^+^/PCNA^+^ pRG lining the tectal ventricle at 3-dpl. White box in **(i)** denotes images shown in **(j** and **k)**. **(l)** Total EdU^+^/GFAP^+^ proliferating radial-glial (pRG) cells arising from activated qRG at times post-lesion. One-way ANOVA; F (4, 22) = 4.479, *p* = 0.0085; Tukey’s multiple comparisons test: 3-dpl vs control, *p* = 0.0308; 3-dpl vs 12-hpl, *p* = 0.0133; 3-dpl vs. 1-dpl, *p* = 0.0707; 3-dpl vs 7-dpl, *p* = 0.0308. **(m)** Fraction of EdU^+^/GFAP^+^ cells as a percentage of the total EdU^+^ population in the PGZ at 1-dpl and 3-dpl. **(n**,**o)** Double-labelling of PCNA with the pan-neuronal marker HuC/D demonstrating an absence of PCNA^+^/HuC/D^+^ cells under control condition (**n**) and at 3-dpl (**o,p**) in the densely populated neuronal layer (Neu-L). Co-labelling of PCNA and basic lipid binding protein (BLBP) can be observed as previous in the qRG-layer (qRG-L; yellow arrow). Experimental replicates were combined for all statistical analyses. All data presented are mean ± S.E.M. Significance was accepted at *p* < 0.05 and is denoted by an asterisk. In panels b,e,h,i,n,p DAPI nuclear counterstaining (blue) was performed. In all cross-sectional images dorsal is oriented up. hpl, hours post lesion; dpl, days post lesion.

How populations of qRG and progenitors along the NE lineage modulate their proliferative behaviour upon lesion remains poorly understood. To examine the proliferative response of qRG following lesion, we performed EdU-labelling in the *Tg(gfap:GFP)*^*mir2001*^ reporter line between 12-hours post lesion (hpl) and 7-dpl (Fig. 3a; control, *n* = 5; 12-hpl, *n = *5; 1-dpl, *n* = 8; 3-dpl, *n* = 5; 7-dpl, *n* = 4). Using 4-hr EdU-chase periods we observed a progressive increase in the proliferative response in the PGZ (qRG-L + Neu-L) that peaked at 3-dpl before significantly decreasing at 7-dpl (Fig. 3b,c). The same trend in EdU-labelling as observed in the entire PGZ was seen within the quiescent RG layer (qRG-L) of the tectum, with a significant increase in the EdU population size at 3-dpl compared with all other groups (Fig. 3d). Importantly, from the qRG layer we successfully identified a subset of qRG that newly entered the cell cycle and were EdU^+^/gfap:GFP^+^ (Fig. 3e–h). We further confirmed this finding using PCNA, illustrating an even greater number of newly proliferative co-labelled RG cells (Fig. 3i–k). Analysis of the EdU^+^/gfap:GFP^+^ population showed that these proliferating RG, hereafter referred to as “pRG”, were restricted to a 48-hr window spanning 1-dpl through to 3-dpl. The presence of pRG were maximal at 3-dpl and significantly different from most other groups (Fig. 3l). Interestingly, despite an approximate doubling of the number of pRG cells from 1-dpl to 3-dpl, the fraction of pRG cells compared to the total EdU population remained steady at 25% (Fig. 3m). Finally, to examine the possibility that neurons near the lesion site may undergo dedifferentiation and contribute to the generation of newborn neurons, we stained for PCNA in combination with HuC/D and basic lipid binding protein (BLBP) in control animals and at 3-dpl (*n* = 3). Compared to control animals where, as expected, no PCNA-labelling was observed in tectal layers (TeO, Neu-L, qRG-L; Fig. 3n; also see Fig. 1a), at 3-dpl PCNA^+^ cells were detected mainly in the qRG-layer with no evidence of double-labelling with the pan-neuronal marker HuC/D in the densely packed neuronal layer or more superficial tectal layers (Fig. 3o,p). Consistent with our previous experiments, we documented co-expression of the glial marker BLBP with PCNA in the qRG-layer at 3-dpl (yellow arrow in Fig. 3o,p). These experiments highlight the ability of a subpopulation of qRG to be stimulated to enter a proliferative state as a result of physical injury.

**Figure 4:**
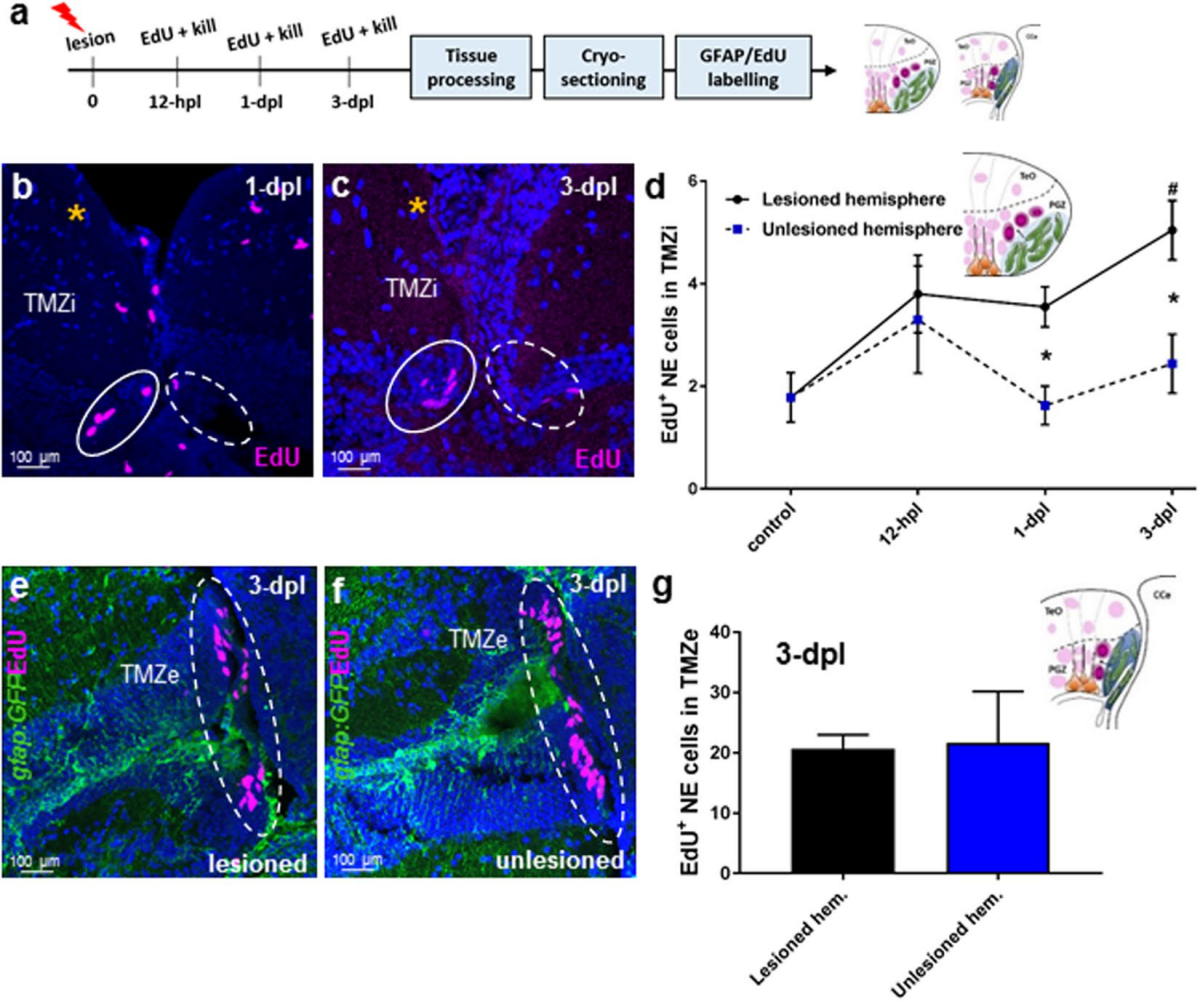
Cell proliferation post-lesion in the TMZi (stem cell zone 2) and TMZe (stem cell zone 3). **(a)** Experimental design to investigate EdU proliferation by NE progenitors of the TMZi and TMZe. **(b,c)** Examples of the EdU^+^ population size of NE amplifying progenitors (pink EdU) in the TMZi observed in the lesioned (yellow asterisk; solid white circle) and unlesioned (dashed white circle) hemispheres at 1-dpl (**b**), and 3-dpl (**c**). **(d)** Number of EdU^+^ NE cells in the TMZi of lesioned hemispheres at times post-injury compared with control (uninjured animal) levels (one-way ANOVA, F (3, 18) = 7.686, *p* = 0.0016; Tukey’s multiple comparisons test: control vs 3-dpl, *p* = 0.005; significance denoted by^#^), and between lesioned and unlesioned hemispheres at each time point (unpaired t-test, two-tailed: 1-dpl lesioned vs 1-dpl unlesioned, *p* = 0.0061; 3-dpl lesioned vs 3-dpl unlesioned, *p* = 0.0096; significance denoted by *). **(e**,**f)** Examples of EdU^+^ labelling (pink) of NE cells in the TMZe of the posterior tectum (white dashed circles) at 3-dpl between the lesioned and unlesioned hemispheres. **(g)** Quantification of the number of EdU^+^ cells between the lesioned and unlesioned hemispheres in the TMZe (unpaired t-test, two-tailed: *p* = 0.9211). Experimental replicates were combined for all statistical analyses. All data presented are mean ± S.E.M. Significance was accepted at *p* < 0.05 and is denoted by an asterisk unless stated otherwise. In panels b,c,e,f DAPI nuclear counterstaining (blue) was performed. In all cross-sectional images dorsal is oriented up. TMZi, internal tectal marginal zone; TMZe, external tectal marginal zone; hpl, hours post lesion; dpl, days post lesion.

Analysis of NE amplifying progenitors at the TMZi (control, *n* = 8; 12-hpl, *n* = 3; 1-dpl, *n* = 5; 3-dpl, *n* = 6) and more slowly cycling NE progenitor of the TMZe (3-dpl, *n* = 4) showed that tectal injury had differential effects on the proliferative behaviour of these two progenitor pools (Fig. 4a). We detected a significant increase in EdU^+^ cells at 1-dpl and 3-dpl between lesioned (solid circles in Fig. 4b,c; solid line in Fig. 4d) and unlesioned (dashed circles in Fig. 4b,c; dashed line in Fig. 3d) hemispheres in NE amplifying progenitors at the TMZi (Fig. 4d; denoted by *). Additionally, a significant increase between homeostatic levels of NE amplifying progenitors and the lesioned hemisphere at 3-dpl was observed (Fig. 4d; compare control vs. 3-dpl for lesioned hemisphere; denoted by#). Conversely, rates of cell division remained unchanged in the NE progenitors of the TMZe between lesioned and unlesioned hemispheres when quantified at 3-dpl (Fig. 4e–g; 3-dpl, *n* = 4). These results imply that injury acts specifically on NE amplifying progenitors proximal the lesion site, rather than the mixed NE lineages of stem and progenitor cells that compose the TMZe^54^. Given these findings, we specifically targeted the qRG population and NE amplifying progenitor pool of the TMZi in subsequent experiments.

### Progeny of proliferative radial-glia produce only newborn glia, whereby neurogenic output from neuro-epithelial-like amplifying progenitors is enhanced following lesion

**Figure 5:**
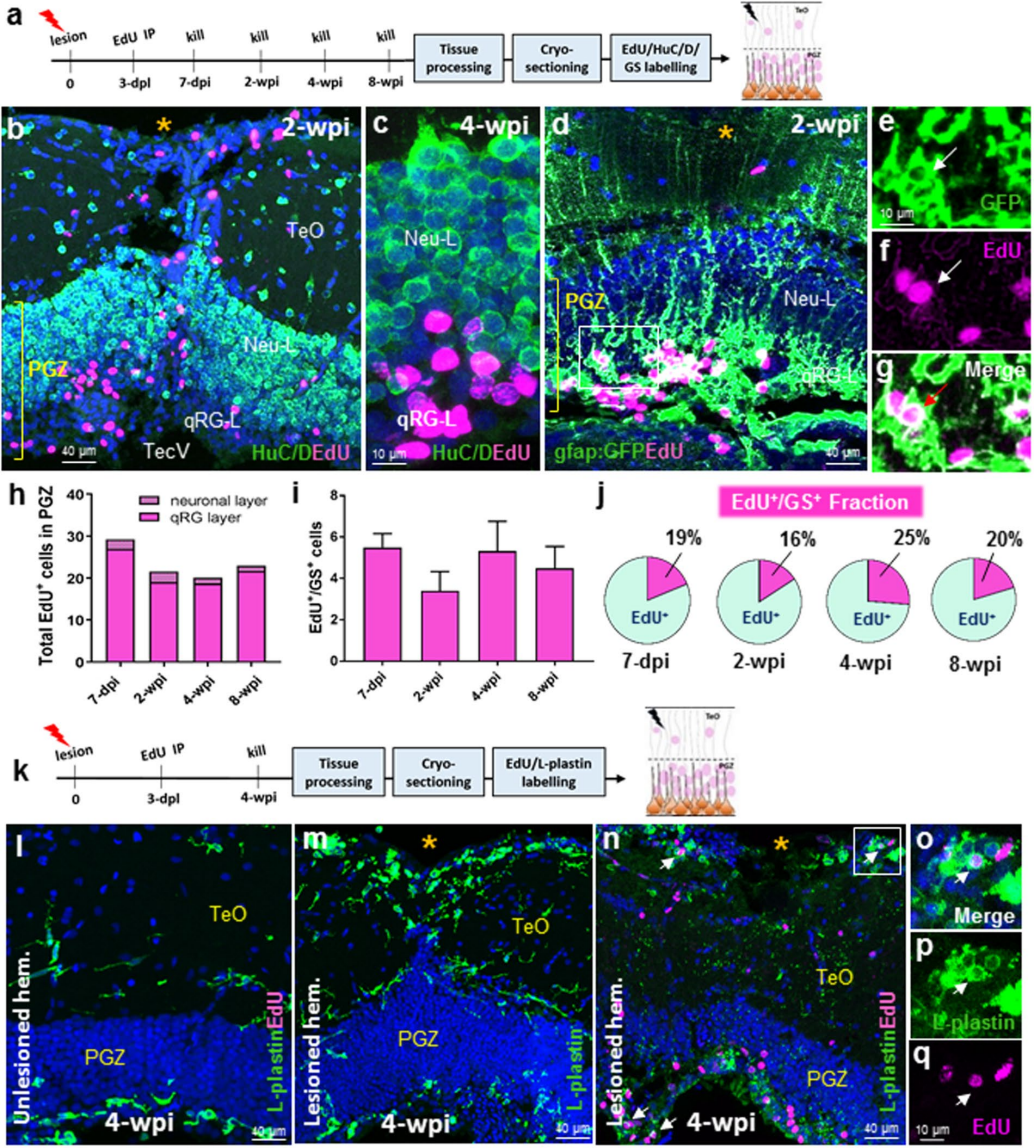
Differentiation post-lesion in activated qRG of the qRG-layer. **(a)** Experimental design to study differentiation of proliferating radial-glia (pRG) at chase periods post-EdU injection. **(b**,**c)** Low and high magnification examples displaying the absence of co-labelling of EdU^+^ cells (pink) in the PGZ with the neuronal marker, HuC/D (green) at 2-wpi (**b**) and 4-wpi (**c**). **(d**–**g)** Representative images of co-labelling of EdU^+^/gfap:GFP^+^ cells in the qRG layer (qRG-L) of the PGZ at 2-wpi. White box in (**d**) shown at higher magnification and in separate channels confirming co-labelling of pRG with EdU (**e**–**g;** white arrows). **(h)** Total EdU^+^ cells in the upper neuronal layer and deeper qRG layer at consecutive chase periods (one-way ANOVA; F (3, 35) = 2.239, *p* = 0.1009; Tukey’s multiple comparisons test: 7-dpi vs 2-wpi, *p* = 0.2082; 7-dpi vs 4-wpi, *p* = 0.1072; 7-dpi vs 8 wpi, *p* = 0.3333; 2-wpi vs 4-wpi, *p* = 0.9863; 2-wpi vs 8-wpi, *p = *0.9984; 4-wpi vs 8-wpi, *p* = 0.9621). Standard error of the mean for total EdU^+^ cells (neuronal layer + qRG layer): 7-dpi ± 2.4; 2-wpi ± 2.9; 4-wpi ± 2.7; 8-wpi ± 3.5. **(i)** Quantification of EdU^+^/GS^+^ cells at increasingly longer chase periods post tectal lesion in the qRG layer (one-way ANOVA; F (3, 17) = 0.7972, *p* = 0.5123; Tukey’s multiple comparisons test: 7-dpi vs 2-wpi, *p* = 0.5190; 7-dpi vs 4-wpi, *p* = 0.9994; 7-dpi vs 8- wpi, *p* = 0.9232; 2-wpi vs 4-wpi, *p* = 0.5845; 2-wpi vs 8-wpi, *p = *0.9107; 4-wpi vs 8-wpi, *p* = 0.9533). **(j)** Fraction of EdU^+^/GS^+^ cells as a percentage of the total EdU^+^ population in the PGZ at four chase periods examined. **(k)** Experimental design to examine the contribution of macrophages, using the L-plastin antibody (green), to cell proliferation at the site of injury at 4-wpi. **(l)** The unlesioned hemisphere displays a small number of non-proliferative resident microglia in the TeO and PGZ. **(m)** A large population of amoeboid-like macrophages are observed in the TeO surrounding the tectal lesion site, with L-plastin staining in the PGZ resembling the unlesioned condition. Co-labelling with EdU (pink) reveals a population of L-plastin^+^ cells that are in a proliferative state (white arrows). White box is shown in higher magnification in panels o,p. **(o**,**p)** Merge and single channels showing a representative L-plastin^+^/EdU^+^ cell (white arrow) adjacent the lesion site at 4-wpi. Experimental replicates were combined for all statistical analyses. All data presented are mean ± S.E.M. Significance was accepted at *p < 0.05. Orange asterisk (*) denotes the lesion site in (**b**,**d**,**m**,**n**). In panels b–d, l–o, DAPI nuclear counterstaining (blue) was performed. In all cross-sectional images dorsal is oriented up. dpl, days post lesion; dpi, days post EdU injection; wpi, weeks post EdU injection.

Successful neuro-regeneration of brain tissue requires that adult stem cells have the capacity to give rise to newborn neurons over time. To first test if pRG progenitors were capable of reparative neurogenesis, we injected zebrafish at 3-dpl with EdU, and provided chase periods ranging from 7-days post injection (dpi) to 8-weeks post injection (wpi; Fig. 5a; 7-dpi, *n* = 6; 2-wpi, *n* = 5; 4-wpi, *n* = 6; 8-wpi, *n* = 5). Across all chase periods, we were unable to find evidence of newly co-labelled EdU^+^/HuC/D^+^ cells in the PGZ in proximity to the lesion site that could have arisen from the pRG population (Fig. 5b-,c). Given this result, we asked whether pRG may instead undergo gliogenesis to replace damaged RG following lesion within the qRG layer of the PGZ. In agreement with this hypothesis, we consistently identified a population of co-labelled EdU^+^/GS^+^ cells within the qRG layer following all chase periods (Fig. 5d–g). Quantification revealed no significant difference in the EdU^+^ population size throughout the PGZ (Fig. 5h; neuronal layer + qRG layer), nor in the newborn EdU^+^/GS^+^ population (Fig. 5i) at all time points following EdU injection. By taking the ratio of co-labelled cells to the total EdU^+^ population we found that this cell fraction did not deviate by more than 10% across groups (Fig. 5j). These findings propose that pRG are unipotent in the tectum after stab lesion, and function to produce radial-glia, to replenish the qRG cells lost to injury.

While investigating the differentiation potential of pRG, we observed a number of EdU^+^ cells in both the superficial tectal layers (TeO) and PGZ that were not accounted for by previous marker combinations. To examine whether, similar to short time periods post-injury (see Supplementary Fig. S4), immune cell proliferation may continue to take place up to 1-month post-lesion, we injected a separate cohort of animals with EdU at 3-dpl, and chased until 4-wpi (Fig. 5k; *n* = 3). In the unlesioned hemisphere, double-labelling of EdU along with the pan-leukocyte marker, L-plastin, showed the same pattern of macrophage distribution as uninjured controls (Fig. 5l and see Supplementary Fig. S4e). At the site of injury in the lesioned hemisphere, a considerable population of L-plastin^+^ blood-born leukocytes and resident microglia were detected in the superficial tectal layers, with fewer cells observed within the qRG layer of the PGZ at 4-wpi (Fig. 5m). Of the L-plastin^+^ cells detected, a subpopulation was found to be L-plastin^+^/EdU^+^ in both the TeO and qRG layer of the PGZ populations (Fig. 5n–q), explaining a proportion of the outstanding EdU^+^ population post-injury. These findings indicate that even at late time points following tectal injury, immune cells continue to proliferate to maintain the required population needed for tissue recovery.

**Figure 6:**
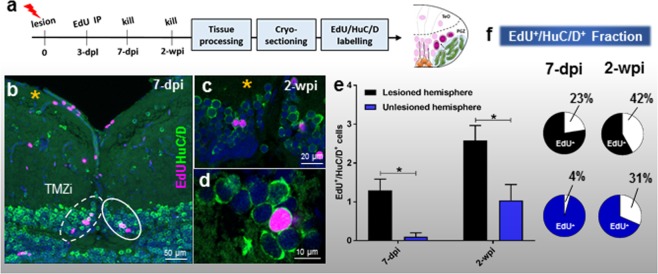
Differentiation post-lesion in NE amplifying progenitors in the TMZi. **(a)** Experimental design to investigate the time course of differentiation of NE amplifying progenitors to newborn neurons at 7-dpi and 2-wpi. **(b)** Image displaying the tectal midline showing the TMZi of lesioned (yellow asterisk; white dashed circle) and unlesioned (solid circle) hemispheres at 7-dpi. **(c**,**d)** High magnification examples showing co-labelling of EdU^+^/HuC/D^+^ cells adjacent the TMZi at 2-wpi. **(e)** Number of EdU^+^/HuC/D^+^ cells between the lesioned and unlesioned hemispheres at 7-dpi and 2-wpi of EdU (unpaired t-test, two-tailed: 7-dpi lesion vs 7-dpi unlesioned, *p* = 0.0083; 2-wpi lesion vs 2-wpi unlesioned, *p* = 0.0336). **(f)** Fraction of EdU^+^/HuC/D^+^ cells as a percentage of the total EdU^+^ population in the PGZ across chase times post-lesion. Experimental replicates were combined for all statistical analyses. All data presented are mean ± S.E.M. Significance was accepted at *p < 0.05. In panels b-d, DAPI nuclear counterstaining (blue) was performed. In all cross-sectional images dorsal is oriented up. TMZi, internal tectal marginal zone; dpl, days post lesion; dpi, days post EdU injection; wpi, weeks post EdU injection.

We next investigated whether increased proliferation of NE amplifying progenitors of the TMZi led to elevated rates of neurogenesis at one and two weeks post-injury (Fig. 6a; 7-dpi, *n* = 6; 2-wpi, *n* = 5). Within the TMZi we identified a significant increase in the co-labelled population of EdU^+^/HuC/D^+^ cells, with more than double the number of EdU^+^/HuC/D^+^ cells by 2-wpi (Fig. 6b–e). This observation was further supported by the enlarged fraction of the co-labelled population in the lesioned hemisphere (percentage of white:black in circles) between time points (Fig. 6f). These results illustrate that NE amplifying progenitors of the TMZi of the adult zebrafish increase their constitutive rate of neurogenesis following tectal lesion.

### Physiological levels of Wnt/β-catenin signalling are unaltered in the qRG layer, but increased in the TMZi following injury

**Figure 7:**
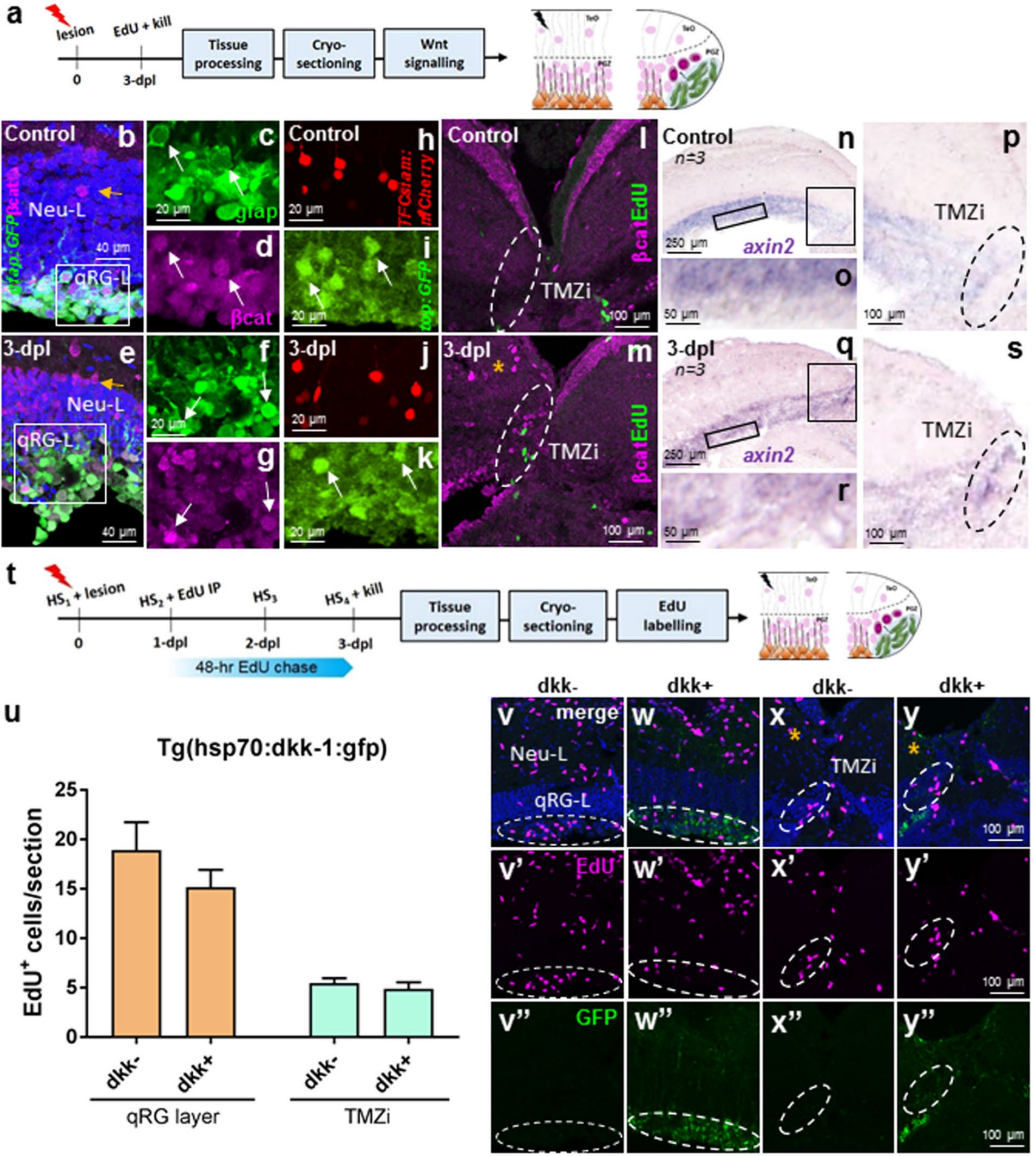
Wnt/β-catenin signalling in stem cell zone 1 (qRG-L) and zone 2 (TMZi) following tectal lesion. **(a)** Experimental design to study Wnt/β-catenin signalling at 3-dpl. **(b–d)** Physiological levels of β-catenin expression in the neural layer (Neu-L) and qRG layer (qRG-L; stem cell zone 1). A small number of β-catenin-positive neuronal cell bodies are seen in the Neu-L (yellow arrow). White box depicts higher magnification images of the qRG-L in (**c**,**d**). Split-channel images showing co-labelling of qRG (**c**) and β-catenin (**d**; white arrows). **(e**–**g)** Lesion-induced β-catenin staining in the neural layer (Neu-L) and qRG layer (qRG-L) showing upregulation in the proportion of neuronal cells expressing nuclear β-catenin. White box depicts higher magnification images of the qRG-L in (**f**,**g**). Split-channel images showing co-labelling of qRG (**f**) and β-catenin (**g**; white arrow). **(h**–**k)** Common expression pattern of Wnt activity observed in the qRG-L between control (**h**,**i**) and at 3-dpl (**j**,**k**) using the Wnt reporter lines *Tg(TCFSiam:mCherry)* and *Tg(top:GFP*; white arrows). **(l**,**m)** β-catenin expression and EdU-labelling at the TMZi (stem cell zone 2; dashed white circles) under control conditions (**l**) and at 3-dpl (**m**, orange asterisk), showing increased nuclear expression in putative neurons post-lesion. **(n**–**p)** Homeostatic levels of *axin2* expression in the qRG layer (**o**) and TMZi (**p**; black dashed circle). Black boxes in (**n**) denote higher magnifications in (**o**,**p**). **(q**,**s)** *axin2* expression 3-dpl in the qRG layer (**r**) and TMZi (**s**; black dashed circle). Black boxes in (**q)** denote higher magnifications in (**r**,**s**). **(t)** Experimental design to study the requirement of Wnt/β-catenin signalling for the proliferative response of stem cells to tectal stab lesion using the heat-shock line *Tg(hsp70l:dkk-1:gfp)*. **(u)** EdU population size in the qRG layer and TMZi shows no change in proliferation post-injury in the absence of Wnt signalling (dkk+) compared with wildtype animals (dkk-) using the *Tg(hsp70:dkk-1:gfp)* transgenic line (unpaired t-test, two-tailed: qRG layer, *p* = 0.3081; NE-Ap zone, *p* = 0.4960). **(v-**w**”)** Representative images of EdU^+^ staining (pink) in the qRG layer (dashed lines) in dkk- (control; **v-**v”) and dkk + **(w-**w**”**) post-lesion. **(x-**y”) Representative images of EdU^+^ staining (pink) in the TMZi (dashed lines) in dkk- (control; **x-**x**”**) and dkk + **(y-**y**”**) post-lesion. Orange asterisk denotes the lesioned hemisphere. Note GFP^+^ expression observed in the dkk + (w”,y”) but not dkk- (v”,x”). Experimental replicates were combined for all statistical analyses. All data presented are mean ± S.E.M. Significance was accepted at *p < 0.05. In panels b,e, v–y, DAPI nuclear counterstaining (blue) was performed. In all cross-sectional images dorsal is oriented up. TMZi, internal tectal marginal zone; dpl, days post lesion.

Wnt/β-catenin signalling has repeatedly been shown to be essential for vertebrate midbrain-hindbrain development^69–72^ as well as tissue regeneration within the CNS^43,44,73,74^, and non-CNS structures of the zebrafish^75–78^. To investigate the role of Wnt/β-catenin signalling in the cellular response of qRG and NE amplifying progenitors following tectal lesion, we analysed the expression pattern of nuclear β-catenin expression and the downstream target gene, *axin2*, of the canonical Wnt pathway under physiological conditions compared with 3-dpl (Fig. 7a). Using antibodies against β-catenin, we report that baseline levels of nuclear β-catenin expression within qRG cells is widespread across the qRG layer (Fig. 7b–d; see Supplementary Fig. S5a–d). However, at 3-dpl quantification showed no significant difference in the number of gfap:GFP^+^/βcat^+^ cells detected compared with control (Fig. 7e–g; see Supplementary Fig. S5e–I; *n* = 4 control, *n* = 3 lesion). High magnification images of single- and multi-channel immunostaining of individual qRG further demonstrated that the nuclear expression of β-catenin under both homeostatic (i.e. control) and lesioned conditions also remained unchanged (see Supplementary Fig. S5a,h high magnification insets). Repeating these experiments using established transgenic Wnt reporter lines, such as *Tg(top:GFP)*, a Wnt reporter line containing 4 TCF binding sites upstream of a destabilized GFP transgene^79^, and *Tg(TCFSiam:mCherry)*, a Wnt reporter containing 7 multimerized TCF/LEF binding sites and the *Xenopus* Siamois promoter upstream of mCherry^80^, recapitulated this same pattern in the qRG layer (Fig. 7h–i, control; Fig. 7j,k, 3-dpl). Interestingly, we detected a clear increase in the proportion of mature neurons in the superficial PGZ expressing nuclear β-catenin following lesion (Fig. 7b,e, yellow arrows). Examination of the TMZi, composed of NE amplifying progenitors along with adjacent neurons of the upper neuronal layer of the PGZ, additionally revealed a small population of putative β-catenin^+^ neurons post-injury proximal to EdU^+^ NE amplifying progenitor cells (Fig. 7l,m; dashed circle). Despite the above, no evidence of nuclear β-catenin staining within individual EdU^+^ NE amplifying progenitors under control or lesioned conditions was observed in the TMZi (Fig. 7l,m; green cells). Further examination of the downstream Wnt target gene, *axin2*, in the quiescent RG layer and TMZi confirmed the same pattern of expression between these two stem/progenitor tectal domains (Fig. 7n–p, control; Fig. 7q,s, 3-dpl). These findings show that while Wnt/β-catenin signalling in qRG following lesion appears unaltered, Wnt signalling is elevated in the TMZi post-injury where NE amplifying progenitors reside and respond to tectal injury.

### Wnt/β-catenin signalling is not required for stem/progenitor proliferation post-injury

To test whether homeostatic levels of Wnt/β-catenin signalling were required for injury-induced proliferation of cells within the qRG layer and TMZi, we next over-expressed Dkk-1, a negative regulator of Wnt signalling over 4-consecutive days using the *Tg(hsp70:dkk-1:gfp)* transgenic line^75^. We subsequently quantified cell proliferation following a 48-hr EdU chase compared with wildtype levels (dkk-) in the lesioned hemisphere (Fig. 7t; wildtype, *n* = 4; dkk + , *n* = 4). Surprisingly, we detected no significant change in the population size of EdU^+^ cells between dkk- and dkk + fish in either stem cell zone analysed (Fig. 7u–y). Taken together, these experiments imply that the proliferative responses within distinct stem/progenitor niches following tectal injury appear to arise independent of Wnt/β-catenin signalling.

## Discussion

**Figure 8:**
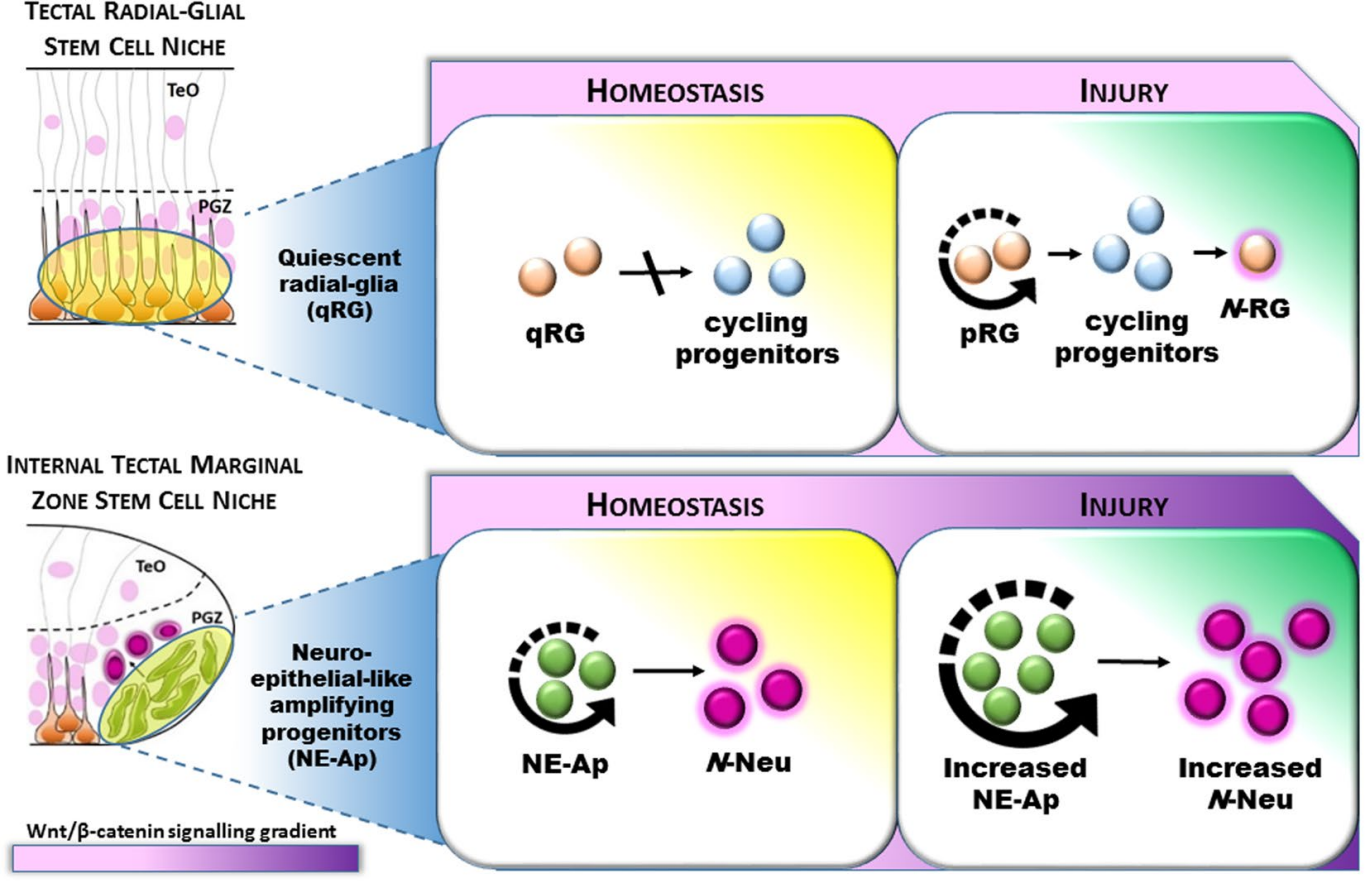
Model displaying the response of niche-specific stem/progenitor populations following lesion to the adult optic tectum. Top**:** Quiescent radial-glia (qRG) are unable to produce cycling progenitors under homeostasis at the tectal ventricle. Post-injury, a subpopulation of qRG transition to a proliferative state (pRG) to produce newly cycling progenitors which differentiate into *de novo* radial-glia (*N-*RG). Bottom: Neuro-epithelial-like amplifying progenitors (NE-Ap) are constitutively active under homeostasis, acting as a lifelong source of newborn neurons (*N*-Neu) at the internal tectal marginal zone (TMZi). Tectal injury induces NE-Ap to increase their homeostatic level of cell proliferation, leading to an increase in neuronal output to replace neurons lost to damage. This regenerative process is associated with an increase in Wnt/β-catenin signalling. TeO, tectum opticum; PGZ, periventricular grey zone.

Our findings support the notion that niche-specific stem/progenitor cells in the adult zebrafish brain are distinguished by discrete regenerative capacities. We show that both quiescent and active stem/progenitor populations of the adult tectum play an important role in repairing damaged tissue following injury. To date, it has been unclear whether the homogeneous quiescent radial-glial (qRG) population that composes the roof of the midbrain ventricle retain a regenerative program to repopulate lost neurons and glia following tectal injury. Our data highlight that lesion-induced, newly proliferating radial-glia (pRG) of the tectal periventricular grey zone (PGZ) are unipotent, producing progeny destined for a radial-glia fate (Fig. 8 – top). Conversely, we report that constitutively active neuro-epithelial-like amplifying progenitors of the internal tectal marginal zone (TMZi) that contribute to lifelong neurogenesis, additionally respond to injury by increasing their normal rate of proliferation and neurogenesis alongside elevated levels of Wnt/β-catenin signalling (Fig. 8 - bottom). The balance by which quiescent versus constitutively active stem/progenitor cells respond to neuro-trauma in the CNS remains poorly understood. Such diversity in the injury response of niche-specific stem and progenitor cells has been documented in the hippocampal dentate gyrus^81,82^ and subventricular zone (SVZ) of the adult mammalian brain^83^. The results of our study reflect our earlier work examining neurogenic plasticity in stem cell compartments of the adult zebrafish brain, where we reported that upon changes to the social environment^36^ or sensory stimuli^37^, NE cells appear more intrinsically primed to modulate their cellular behaviour compared with RG. Understanding the molecular differences between the newly identified, slowly-cycling *Her5*-positive NE stem cells in the TMZe^52–54,62,84^ and qRG of the midbrain periventricular zone (PGZ), along with actively cycling RG in the telencephalon would further aid to determine how quiescence and proliferative activity is controlled.

Vertebrate neural stem cell niches are characterized by heterogeneity in the stem/progenitor population^5–15,31^. Accumulating evidence shows that such heterogeneity also may exist amongst quiescent stem and progenitor cell populations. Single-cell analyses of quiescent neural progenitors in both the SVZ and hippocampus show that subpopulations within a niche can be distinguished by diverse characteristics such as their response to hormones, brain ischemia, metabolic state, and growth factors^29,30,81,85^. This indicates that different quiescent precursors are likely governed by varying degrees of plasticity. Morphological analysis has also revealed that a subpopulation of quiescent B1 cells of the adult ventricular-SVZ are defined by the presence of envelop-limited chromatin sheets, uniquely discriminating this population within the forebrain neurogenic niche^86^. Across adult zebrafish neurogenic zones, niches of the forebrain dorsal telencephalon and hindbrain vagal lobe are exemplary of domains dominated by heterogeneous RG stem/progenitor populations^25,37^. The majority of the RG population in these niches reside in a non-cycling state under homeostasis, with only a small number of cells taking up proliferative markers with short pulse-chase experiments. With injury to the forebrain however, a significant proportion of qRG re-enter the cell cycle^20,21,24,46,47^. Impressively, these activated RG in the telencephalon appear to possess the molecular code to repopulate the full complement of lost neuronal lineages^20^.

The RG tectal stem cell niche composes the entire roof of the midbrain ventricle and displays lifelong quiescence apart from RG that are thought to act as amplifying progenitors in proximity to NE amplifying progenitors of the TMZi^34,54^. The tectal population of qRG therefore contrasts the mixture of proliferative and non-proliferative RG observed in the forebrain and hindbrain. In this study we proposed that the qRG population may be endowed with the intrinsic program for neuroregeneration, however our evidence points towards this population as serving a more structural role in maintaining the epithelial barrier and supporting the neuronal circuitry. In agreement, our data shows limited production of newborn RG progeny from pRG (Fig. 5i), along with the lack of neurons produced locally to replace those damaged in the upper neuronal layer of the PGZ. Interestingly, the qRG display similar characteristics of ependymal and astroglial lineages in the mammalian brain – e.g. proliferate but remain within their own lineage. In the future it will be important to compare the molecular characteristics of the qRG with the mammalian glial cell lineages.

Shimizu *et al*.^87^ recently showed that injury to the caudal tectum of zebrafish resulted in a very low abundance of cells labelled with BrdU/HuC/D in the PGZ, proposing that neurons may possibly be produced after injury. It is unclear if the produced neurons survive long-term to make a functional contribution to neuroregeneration, since they were analysed at only a short time after injury (1-week). Production of neurons at the lesion site in the PGZ is in contrast to our findings, as we did not detect EdU-labelled neurons in the vicinity of the lesion or central parts of the tectum after examining short and long-term time-points (i.e. 7-dpi, 2-wpi, 4-wpi, 8-wpi, *see* Fig. 5). Therefore, our labelling experiments suggest that no neurons were regenerated anew at the lesion site. The reason for the observed difference may be related to age of fish, size and placement of injury, and long-term survival of cells. Shimizu and colleagues employed young adult zebrafish and placement of lesions appeared to be located closer to the TMZi. Rather, we used older and larger adults (≥6 months) during our experiments, and the placement of injury was consistently in the central part of the tectal hemisphere, spatially distant from the TMZi. Using label retention assays and lineage tracing it has been shown that the tectal RG transiently act as glial and neural progenitors once they emerge from the TMZ^54^. Therefore, injury and responding RG close to the TMZ may display enhanced plasticity and act as bi-potent progenitors and may explain observed differences between our work and that of Shimizu *et al*.^87^. Age-related changes in the ability of tectal RG to be induced to take part in the regenerative process may also contribute, since differences in the potential of RG-like progenitors in the cerebellum change with age and impact regeneration^51^. Our results suggests that the qRG mainly, or perhaps even only, contribute to maintaining the structural integrity of the ventricular epithelium towards the tectal ventricle after injury in the adult zebrafish. In attempt to elaborate on this we performed genetic lineage tracing using a tamoxifen-inducible Cre driver within the RG lineage using the transgenic line *Tg(her4*.*3:mCherry-T2A-CreERT2)*^20^. The recombination after injury was low and mosaic preventing quantitative and conclusive results. However, the limited data obtained from these experiments supported that RG keep to their lineage after injury. Additional lineage tracing experiments in juvenile and adult zebrafish are required to conclusively address the potency of the qRG population in the tectum following injury. Nevertheless, even if there is low production of neurons in the PGZ after injury the amount seems too small to play a pivotal role in neuroregeneration and functional recovery.

A particularly exciting finding of our study was that NE amplifying progenitors underwent elevated rates of constitutive proliferation and neurogenesis following lesion to the midbrain nearly ~350 µm from the TMZi. In the uninjured context, NE amplifying progenitors function to circumferentially add newborn neurons to the tectum to maintain tissue growth^34^. Our data indicate that signals initiated at the lesion site are received by these NE progenitors of the lesioned hemisphere, leading to increases in both cell proliferation (Fig. 4d) and neurogenesis (Fig. 6e) beyond changes detected in the unlesioned hemisphere. Work by Shimizu *et al*.^87^ using the broader cell cycle marker, Proliferating Cell Nuclear Antigen (PCNA), reported little change in the constitutively active NE amplifying progenitor population of the TMZ after tectal stab injury. This is surprising, as we readily observed detectable changes in cell proliferation between lesioned and unlesioned hemispheres using the *S*-phase marker EdU. While both our study and that of Shimizu *et al*.^87^ examined the size of the NE population over a similar experimental timeline post tectal injury, the region of analysis was markedly different. This likely explains the disparity between the results of NE experiments presented. We directly quantified cycling NE amplifying progenitors at the TMZi at the same level as the lesion canal, whereby Shimizu and colleagues’ quantification included NE cells spanning the TMZi and TMZe. Therefore, the analysis encompassed a mixture of both NE amplifying progenitors and slowly cycling label-retaining NE cells^54^, likely diluting the ability to detect a change in the NE population between the lesioned and unlesioned hemispheres. This explanation is corroborated by our analysis within the TMZe that showed no significant difference in the number of proliferating NE cells between hemispheres post-injury (*see* Fig. 4e–g).

Our findings argue the regenerative mode of NE amplifying progenitors in the TMZi and whether it may be viewed as compensatory neurogenesis, rather than lineage-directed neural regeneration. Compensatory neurogenesis following CNS damage generally repairs and strengthens the circuitry without the necessity to directly replace lost cells and could therefore be seen as a broader restorative mechanism^33^. In line with our data, as well as previous work in the telencephalon^20^, increases above constitutive levels of cell proliferation and/or neurogenesis normally produced from a stem cell niche have also been associated with a compensatory response. Mammalian studies of disease and injury have commonly shown the occurrence of compensatory proliferation/neurogenesis as an endogenous response to CNS tissue repair^88^. Stab lesions immediately within the zebrafish TMZi would be a fruitful approach to examine the potential of NE amplifying progenitors for regenerative neurogenesis and gliogenesis, and to conclude the specific regenerative mode employed by this progenitor pool.

A common form of neuroregeneration brought to light by comparing both the adult cerebellar and tectal niches is the discovery that NE, rather than RG, appear to drive neuroregeneration in the midbrain and hindbrain alike. RG have often been proposed as the major stem cell population regulating regeneration in vertebrates, however here we provide additional evidence supporting the notion that this is likely not the case. In the injured cerebellum, NE are able to produce all cell lineages that they normally produce under homeostasis^51^. In agreement, we show for the first time that, in the tectum, neuroregeneration from a constitutively active population of NE amplifying progenitors functions similarly to populate the neural lineage that is not replenished by pRG cells. Therefore, in line with NE-driven cerebellar regeneration, midbrain NE amplifying progenitors also make use of their homeostatic neurogenic program.

Amongst the cues controlling the stem cell state, developmental signalling pathways play an important role^16,22,48–50,89–92^. Here, we investigated the role of Wnt/β-catenin signalling in regulating qRG and NE amplifying progenitors during the regenerative response given the importance of this pathway for early midbrain-hindbrain boundary patterning^69–71^ and tissue-wide regeneration in the zebrafish^43,44,72–78^. It has recently been found that Wnt signalling components play a role in constitutive cell division and neuronal differentiation from NE amplifying progenitors. Inhibitors of Wnt, such as IWR1, further suppress proliferation of this progenitor population under homeostasis^55^. By examining nuclear β-catenin staining, downstream Wnt targets such as *axin2*, and promoters of Wnt reporter lines (i.e. TCF/Lef, Siam), we show a baseline level of canonical Wnt signalling in both qRG cells of the tectum and within the TMZi (Fig. 7). We find however, that upon tectal lesion Wnt/β-catenin signalling is upregulated uniquely in the TMZi where NE amplifying progenitors reside (Fig. 7m,s). In contrast, a study using other Wnt reporter zebrafish, 6X tcf/Lef-miniP:d2GFP, showed transient upregulation of GFP reporter expression 2-days following tectal lesion in the qRG-layer, in addition to differential temporal expression of *ascl1a* and *dkk1b* over the first 24-hrs post-injury^87^. These data propose that Wnt/β-catenin signalling may be involved in the regenerative process at earlier time point than examined here. This finding led us to hypothesize whether the increase in cell proliferation from the NE amplifying population with injury was controlled by the Wnt/β-catenin pathway. Silencing Wnt signalling over the first 3-days post-lesion by overexpression of the Wnt inhibitor, *Dickkopf-1*, displayed no significant difference in the cycling population within the qRG layer or the TMZi however (Fig. 7u–y). This suggests that despite elevated levels of Wnt/β-catenin signalling following injury, Wnt may not be required for the regenerative response of the NE amplifying progenitor pool or may be acting in combination with other signalling cascades as illustrated in the zebrafish retina^44^. In agreement, Shimizu *et al*.^87^ showed that pharmacological inhibition of Wnt signalling post-injury using the Wnt inhibitor, IWR1, does not affect cell proliferation at the lesion site compared with DMSO controls. However, double-labelling with PCNA and the glial marker Brain Lipid Binding Protein (BLBP; a.k.a FABP7) did reveal that IWRI treatment leads to a significant reduction in the number of PCNA^+^/BLBP^+^ cells, implicating upregulation of Wnt signalling as a potentially important pathway in the activation of quiescent RG post-injury^87^. Since our evidence has consistently pointed towards a NE-driven regenerative mechanism, we did not perform a parallel experiment in the current study. As such, we cannot dismiss the possibility that Wnt, in part, could regulate the proportion of qRG that transit to a pRG state following lesion. Collectively, our results align with studies of the larval zebrafish hypothalamus showing that partial genetic ablation of hypothalamic RG leads to a net increase in cell proliferation in the absence of Wnt/β-catenin signalling^93^.

Earlier studies of the zebrafish CNS have highlighted the heterogeneous role of Wnt/β-catenin signalling in controlling stem cell behaviour. A pointed example of this is in the zebrafish retina. The retina contains populations of ciliary marginal zone (CMZ) NE cells and quiescent Müller glia reflecting the region-specific organization of the TMZi NE amplifying progenitors and qRG. In this model, Wnt/β-catenin signalling is required to permit normal retinal growth from the CMZ, whereas activated Müller glia depend on these signals to generate neuronal progenitors post-lesion^73^. The zebrafish spinal cord instead depends on the Wnt pathway for both RG-derived neurogenesis and axonal regrowth post transection^74,94^. Additionally, Notch signalling regulates stem cell quiescence in distinct stem/progenitor populations^22,48–50^. Therefore, uncovering how Wnt/β-catenin signalling may associate with Notch signalling in diverse physiological and pathological states would serve well to resolve the contribution of these two heavily studied pathways to developmental and regenerative processes in the zebrafish CNS.

In the present study we have illustrated that tectal stab lesion leads to a dichotomous response in cell proliferation and differentiation between activated qRG and the NE amplifying progenitor population. Our results demonstrate that the qRG in the zebrafish tectum have restricted capability in neuronal repair, highlighting that RG have diverse and distinct functions in the zebrafish brain between neurogenic niches. Furthermore, the results suggest that endogenous stem cell compartments may compensate distally lost tissue by amplifying homeostatic growth. Signals produced as a result of injury are sufficient for these stem and progenitor populations to initiate their individual reparative programs to replace damaged RG and neurons. While the relative contribution and mode of CNS regeneration differs between these adult stem/progenitor populations, our results propose that they complementarily possess the cellular and molecular machinery needed to restore tissue back to its uninjured state over time. Our findings are important in contributing new evidence that the regenerative behaviour of diverse adult stem/progenitor phenotypes contain different intrinsic degrees of regenerative plasticity and regulation in the context of injury. Moving forward, defining the transcriptional profiles of closely related stem/progenitor phenotypes residing in different neurogenic domains under homeostasis and with injury will be fundamental in mapping their distinct molecular programs.

## Methods

### Animals

All zebrafish lines used in experiments were housed in the Monash University FishCore Facility and maintained according to facility regulations. Animals across experiments were of mixed sex and between 6-months to 1-year of age. For individual experiments animals were age and size matched. Upon completion of experiments, zebrafish were sacrificed using an overdose of 0.4% Tricaine (Sigma; E10521) diluted in ice-cold facility water. Experiments were assessed and approved by the Monash University Animal Ethics Committee and were conducted under applicable Australian laws governing the care and use of animals for scientific research.

### Optic Tectum Stab Lesion Assay

Adult fish were anesthetized in 0.04% Tricaine (Sigma; E10521) prior to tectal stab lesion. Stab lesions were performed under a dissecting microscope in the left hemisphere of the tectum using a 30 × ½ gauge canula (Terumo). The cannula was carefully inserted vertically into the centre of the neurocranium overlying the tectum, and slightly anterior to the middle of the tectum (Fig. 1I,j). These landmarks ensured the lesion entered through the tectal layers and into the large, underlying tectal ventricle. Thereafter, fish were returned to experimental tanks and monitored until normal swimming behaviour commenced. Sham fish (controls) were treated similarly, with the exception that the cannula only penetrated the neurocranium, but not further into the brain tissue.

While we found our tectal lesion assay to be highly reproducible, to ensure accurate cell quantification of the lesion response across experiments the following screening criteria had to be met in order for the brain sample to be included in downstream analyses: (1) lesioned hemispheres displayed a clear upregulation of the proliferative marker (EdU or PCNA), and (2) the cannula clearly penetrated the deep PGZ layer as per 4,6-diamidino-2-phenylindole (DAPI) counterstaining.

### EdU Injections

A 10 mM solution of 5-ethynyl-2′-deoxyuridine (EdU; ThermoFisher; A10044) diluted in 1 × -Phosphate Buffered Saline (PBS; pH 7.4) was injected intraperitoneally to label proliferating cells in the cell cycle. The total volume of individual EdU injections was 40 µL.

### Tissue Processing for Immunohistochemistry, Histology, and *in situ* Hybridization

Following sacrifice, brains were exposed by removing the neurocranium and left *in situ* in the head. Zebrafish were subsequently decapitated, and heads then immediately fixed in 2% paraformaldehyde (PFA; Sigma; 158127) diluted in phosphate buffer (pH 7.4) for immunohistochemistry (IHC) or in 4% PFA diluted in 1 × -PBS (pH 7.4) for *in situ* hybridization (ISH) and histology, overnight at 4 °C. The next day, heads were placed in a solution of 8 M ethylenediaminetetraacetic acid (EDTA) for 24-hrs at 4 °C to decalcify surrounding cartilage, before being cryo-embedded in a mixture of fish gelatin (Sigma; G7041) and sucrose (VWR; VWRC0335) in PBS. Heads were sectioned in the cross-sectional plane using a Leica CS3080 Cryostat at a thickness of 18 µm and stored at −80 °C until use.

### Tissue Processing and Whole Brain EdU Staining for Optical Projection Tomography

Tissue processing and whole brain EdU staining on adult wildtype (Tübingen) zebrafish for Optical Projection Tomography (OPT) experiments was completed as described previously^40,65^. Briefly, brains were excised from the neurocranium and immediately fixed in ice-cold 2% PFA overnight. The next day, brains were rinsed over 4-hrs in 1 × -PBS containing 0.3% Triton X-100 (Tx; Sigma; T9284) to remove fixative, then placed in a solution of 1% Tx/5% dimethyl sulfoxide (DMSO; Millipore; 317275) in 1 × -PBS for 24-hrs to increase antibody penetration. Thereafter, brains were incubated in EdU staining mixture for 4-days with gentle agitation, then rinsed before embedding in low melting agarose (Sigma; A9414). Trimmed agarose blocks were dehydrated in pure methanol over 1.5-days, and finally cleared in a 2:1 solution of benzyl benzoate:benzyl alcohol (BABB; Sigma; B6630, 402834).

### EdU Staining on Cryosections

Rehydrated cryosections were stained as per the “Click-iT” reaction between EdU and an azide-modified Alexa dye (Molecular Probes, 2010). Slides were incubated at room temperature in the dark for 30-min in a cocktail consisting of 1 × -PBS, 0.5M L-Ascorbic Acid (Sigma; A5960), 2 M Tris buffer (pH 8.5; Sigma; T6791), Copper II Sulphate (Sigma; C1297), and 100 mM Azide Fluor dissolved in DMSO (555 or 647; ThermoFisher; A20012; A10277). A total volume of 250 µL of staining mixture was used per slide. Counterstaining was performed using DAPI.

### Hematoxylin & Eosin Histology

Hematoxylin & eosin (H&E) histological staining was completed at the Monash Histology Platform at Monash University using a Leica ST5010 Autostainer and CV5030 Coverslipper. Imaging was completed using an Olympus Provis AX70 Widefield brightfield microscope at the Monash Micro Imaging (MMI) facility at Monash University.

### Immunohistochemistry

Immunohistochemistry was performed as previously described^2^. Briefly, rehydrated cryosections were incubated in primary antibody overnight at 4 °C. Tissue was then incubated with secondary antibodies (dilution 1:750) conjugated to Alexa Fluor 488, 555, and 633 (Invitrogen) for 1-hr at room temperature and counterstained with DAPI. For confocal imaging, all sections were mounted in 70% glycerol in 1 × -PBS and cover-slipped.

For HuC/D staining, antigen retrieval was completed by incubating sections in 50 mM Tris buffer (pH = 8.0) for 30-min at 80–85 °C in a tissue incubator. Thereafter, tissue was stained as described previously^25^. For labelling of Proliferating Cell Nuclear Antigen (PCNA), tissue was incubated in 10 mM sodium citrate buffer (pH 6.0) for 15-min at 90 °C.

Primary antibodies used included mouse monoclonal against glutamine synthetase (GS, 1:4000; Millipore; MAB302), rabbit polyclonal against S100β (1:1500; Dako; Z0311), rabbit polyclonal brain lipid binding protein (BLBP; 1:2000; Chemicon; AB9558, now replaced by ABN19), mouse monoclonal against Proliferating Cell Nuclear Antigen (PCNA; PC10, 1:750; Dako; M0879), mouse monoclonal against β-catenin (15B8, 1:1500; Santa Cruz; sc-53483), mouse polyclonal against HuC/D (1:400; Molecular Probes; 16A11), polyclonal rabbit anti-L-Plastin (Lcp1; 1:4000; A kind gift from Michael Brand, Center of Regenerative Therapies Dresden, Germany, Technical University Dresden, Germany), and rabbit polyclonal against Green Fluorescent Protein (GFP, 1:1000; ThermoFisher; A11122).

### Transgenic Lines

The *Tg(gfap:GFP)*^*mi2001*^ line was used to label the quiescent radial-glial (RG) population at the tectal ventricle. *Tg(mpeg1:mCherry)*^*g122*^ was used to label resident microglia and infiltrating macrophages upon lesion, and was a kind gift from Dr. G. Lieschke at the Australian Regenerative Medicine Institute (ARMI). *Tg(top:GFP)*^79^ (also known as: Tg(TOPdGFP), Tg(OTM:GFP)) and *Tg(TFCsiam:mCherry)*^80^ were used to assess canonical Wnt expression. The heat-shock inducible *Tg(hsp70:dkk-1:gfp)*^75^ line was applied to negatively regulate Wnt signalling via *Dickkopf-1* (Dkk-1) with heat-shocks performed as described below.

### *In Situ* Hybridization

*In situ* hybridization on cryosections from adult wildtype zebrafish (Tübingen) was performed as described previously^95^. The *axin2 in situ* probe was generated in our lab from the ZP60 Axin2 plasmid (Addgene #16882) in a pSPORT1 vector, digested with asp718 and transcribed with SP6 RNA polymerase. The probe was used at a concentration of 1:250.

### Heat-Shock Treatments

Adult *Tg(hsp70:dkk-1:gfp)* fish were heat-shocked once daily over four consecutive days to reveal GFP expression and downregulate Wnt/β-catenin signalling. Daily heat-shock treatments were performed in the morning by placing fish into the heat-shock chamber with the water at an initial temperature of 28 °C and the heater turned on to increase the temperature to 38.5 °C over the next hour. Fish were then maintained in the chamber at 38.5 °C for 30-min, before the heater was switched off and the water was progressively returned to 28 °C throughout the remainder of the day. During all heat-shock trials fish were monitored to ensure health was not compromised. Animals negative for the dkk-1 transgene were used as controls and exposed to the same experimental protocol.

### Neuroanatomical Regions of Interest for Imaging and Analysis

#### Periventricular Grey Zone (PGZ) of the optic tectum

The PGZ of the optic tectum, consisting of the upper neuronal layer and deeper quiescent radial-glial layer, was examined between anterior-posterior levels 160–170 as per the adult zebrafish atlas^96^ (Fig. 1c; *stem cell zone 1*). Tissue was investigated specifically at the level in which the lesion occurred, unless stated otherwise. This neuroanatomical domain permitted assessment of changes in the proliferative activity and Wnt expression of qRG populations between lesioned and unlesioned conditions in addition to changes in Wnt expression in neuronal layers.

#### Internal tectal marginal zone (TMZi)

The internal tectal marginal zone (TMZi) was examined at the same anterior-posterior level as the PGZ (i.e. qRG lesion site) to analyze neuro-epithelial-like (NE) amplifying progenitor proliferation, neurogenesis, and Wnt expression between lesioned and unlesioned conditions (Fig. 1c; *stem cell zone 2*).

#### External tectal marginal zone (TMZe)

The external tectal marginal zone (TMZe) of the caudal tectum, consisting of a strip of cells, spanning anterior-posterior levels 208-213^96^ was investigated to assess changes in EdU proliferation (Fig. 1e**;** *stem cell zone 3*), where defined populations of NE parent stem cells, label-retaining NE progenitor cells, and transit amplifying NE progenitors reside^54^.

### Optical Projection Tomography (OPT) Imaging and Analysis

EdU-stained adult zebrafish brains were affixed to metal OPT mounts and imaged using a Bioptonics 3001 OPT scanner (Bioptonics, Edinburgh, UK). For each channel (555 nm; 488 nm) 2-dimensional images were acquired at 0.45° intervals, with each frame averaged twice. EdU-labelling was imaged in the 555 nm channel, while brightfield images of brain volume were captured by imaging auto-fluorescence from the 488 nm channel. Exposure was consistently set at 75% of the maximum signal intensity detected in the brains. 2-dimensional images were then post-processed using nRECON software (Bruker microCT) to yield a final 3-dimensional dataset for visualization and downstream volumetric analysis of changes in EdU using IMARIS software.

To assess the proliferative response within the lesioned hemisphere following stab injury compared with uninjured conditions, we designed an algorithm in IMARIS to calculate the total volume of EdU^+^ labelling in concentric spherical rings radiating outwards from a centre-point (see Supplementary Fig. S1a-b). Here, a centre-point was placed at the junction of the tectal ventricle and qRG-layer where we normally quantify the lesion response (see Supplementary Fig. S1c; yellow dot). From this centre-point, we generated concentric spheres at 50 µm intervals, terminating at 299 µm from the centre-point (i.e. 6 spheres totals; see Supplementary Fig. 1d–l). Our algorithm then calculated the total EdU volume occupied within each consecutive sphere. We predicted that compared to control levels where nearly no cycling cells are observed, following lesion an increase in the total amount of EdU should be detected in one or more spheres. For example, the total EdU volume within the blue sphere (see Supplementary Fig. S1f) ranging from 100–149 µm from the centre-point (see Supplementary Fig. S1c) could be calculated independent of other spheres located more proximal or distal to the lesion site. For normalization, the ratio of EdU volume: sphere volume was calculated, and converted to a proportion (represented as percentage) for statistical analysis and graphical representation.

### Confocal Image Acquisition and Processing

Confocal imaging was completed using a Leica SP5 or SP8 inverted channel confocal microscope with 20X, 40X, 63X, and 100X oil immersion lenses at the Monash Micro Imaging facility (MMI, Monash University). Virtual zoom was applied in cases where cellular detail or co-localization was required. Images shown are maximum projections of z-stacks taken at 1 µm intervals through the z-plane of cryosectioned tissue at 1024^2^ resolution. In all images, dorsal is oriented upwards. For display, brightness and contrast were adjusted using FIJI/ImageJ or LAS AF Lite software.

### Cell Quantification

For all experiments, 2–3 sections at the neuroanatomical region of interest (Fig. 1c,e,h; *stem cell zones 1–3*) in each brain sample were analysed. Quantifications were done by counting cells through the z-stack of confocal images taken at 40X –63X magnification using FIJI/ImageJ or LAS AF Lite software to avoid double counting. For co-labelling experiments, orthogonal views were used to accurately confirm double-positive cells. For each brain section, a sum of the cell population of interest was obtained. Sums from multiple brain sections of a single brain sample were then averaged, with this value representing the average number of cells counted for a single biological specimen.

### Statistical Analysis and Graphical Representation

GraphPad Prism7 was used for all statistical analyses and graphs. Statistics were performed on the average cell counts/section calculated across multiple brain samples. For all experiments, significance was accepted at *p* < 0.05. The Shapiro-Wilk normality test was used to confirm data adhered to a Gaussian distribution. Unpaired t-tests (two-tailed) were used to compare differences between two groups. Comparisons between greater than two treatment groups were completed using one-way ANOVA. For all one-way ANOVA tests, the F-value and exact *p*-values are stated. In cases where a significant between-group effect was present, Tukey’s multiple comparisons tests were performed and the adjusted *p*-value reported. All statistical results, including exact p-values, are reported in figure legends. Graphical data represent mean ± standard error of the mean (S.E.M.). Fractions shown depict the total mean number of co-labelled cells divided by the total mean EdU^+^ population size, and are represented in percentage.

## Supplementary information

Supplementary Figures 1–5

## Data Availability

The datasets generated during and/or analysed during the current study are available from the corresponding author on reasonable request.
