# Supplementary material for: Midbrain tectal stem cells display diverse regenerative capacities in zebrafish
Source: Sci Rep. 2019 Mar 14;9:4420. doi: 10.1038/s41598-019-40734-z (PMC6418144; doi:10.1038/s41598-019-40734-z)

Supplementary Information: 5 Figures, 5 Legends

Midbrain tectal stem cells display diverse regenerative capacities in zebrafish

Benjamin W. Lindsey[1, 2], Georgia E. Aitken[1], Jean K. Tang[1], Mitra Khabooshan[1], Alon M. Douek[1], Celia Vandestadt[1], Jan Kaslin[1*]

[1]*Australian Regenerative Medicine Institute, Monash University Clayton Campus, Clayton, Victoria, 3800, Australia*

[2]*Department of Human Anatomy and Cell Science, College of Medicine University of Manitoba Winnipeg, Canada*

*Correspondence to: jan.kaslin@monash.edu

Supplementary Figure 1. Sphere analysis of cell proliferative following tectal lesion. **(a-b)** Mid-horizontal (**a**) and mid-sagittal (**b**) views of a reconstructed adult brain denoting the sequential, coloured, concentric spherical rings spaced 50 µm apart for analysis of the total EdU volume/sphere volume. **(c)** Image of the centre-point of analysis (yellow circle) positioned at the junction of the quiescent RG layer and the tectal ventricle, overlayed on a control brain (EdU, pink). **(d-i)** Example of increasing sphere distance from centre-point (***see* c**) used for analysis and overlayed on a control brain (EdU, pink). **(j)** Experimental design to investigate the proliferative response using EdU labelling in the lesioned tectal hemisphere at 1-dpl and 3-dpl. **(k)** Proportion of EdU volume/sphere represented as a percentage between consecutive spheres at 1-dpl. Proliferation was greatest at 1-dpl, displaying a progressive reduction in the proportion of EdU volume/sphere towards spheres more distal to the centre-point. A significant different was observed between sphere distance 0-49 µm and 250-299 µm (unpaired t-test, two-tailed: p = 0.036). **(l1-l7)** Reconstructed total volume of EdU occupying consecutive spheres at 1-dpl shown *in situ* across all spheres (**l1*,*** 0-299 µm from centre-point), and additively in overlayed spheres (**l2-l7**). **(m)** Proportion of EdU volume/ sphere represented as a percentage between consecutive spheres at 3-dpl. A significant decrease was observed between the proportion of EdU nearest the centre-point (i.e. 0-49 µm) compared with more distal spheres (one-way ANOVA: F (5, 35) = 3.463, p = 0.0120; Tukey's multiple comparisons test: 0-49 *µ*m vs 50-99 µm, p = 0.0294; 0-49 µm vs 100-149 µm, p = 0.0421; 0-49 µm vs 150-199 µm, p = 0.0441; 0-49 µm vs 200-249 µm, p = 0.0307; 0 – 49 µm vs 250-299 µm, p = 0.0647). **(n1-n7)** Reconstructed total volume of EdU occupying consecutive spheres at 3-dpl shown in situ across all spheres (**n1**, 0-299 µm from centre-point), and additively in overlayed spheres (**n2-n7**). Experimental replicates were combined for all statistical analyses. All data presented are mean ± S.E.M. Significance was accepted at *p < 0.05. With the exception of sagittal views of whole brains shown in panels b, l1, n1, in all other images brains are oriented dorsal up. dpl, days post lesion.

Supplementary Figure 2. Global proliferative response of the midbrain following tectal lesion. **(a)** Experimental design to study the proliferative response using Proliferating Cell Nuclear Antigen (PCNA) immunohistochemistry. **(b)** Cross-sectional view of a control (uninjured) tectum showing a nearly complete lack of $PCNA^+$ labelling in both hemispheres, with the exception of modest proliferation in the valvula cerebelli of the cerebellum (white arrows). **(c)** Cross-sectional view of unlesioned (left) and lesioned (right) tectal hemispheres at 3-dpl illustrating local proliferation at the lesion site (yellow asterisk; white dashed circle), and proliferation in the valvula cerebelli of both hemispheres (white arrows), and along the midline (white box). An increase in ventral hypothalamic proliferation in both hemispheres could be detected at 3-dpl, though this was most pronounced in the lesioned hemisphere (white bracket) compared to the unlesioned hemisphere (red bracket). **(d-f')** Whole brain dorsal and cross-sectional views of the lesioned hemisphere anterior to the lesion site (**d-d'**), at the level of the lesion site (**e-e'**), and posterior to the lesion site (**f-f'**), demonstrating the global proliferative response to tectal injury through the midbrain tectum. In panels **d', e', f',** DAPI nuclear counterstaining (blue) was performed. In all images brains are oriented dorsal up. dpl, days post lesion.TeO, tectum opticum; PGZ, periventricular grey zone; vcb, valvula cerebelli; hyp, hypothalamus.

Supplementary Figure 3. The proliferative response of the adult brain to injury is conserved independent of the site of lesion. **(a-b)** Side-by-side comparison of the proliferative response of the adult brain to telencephalic (EdU, green) and tectal (EdU, pink) stab lesions using OPT imaging to visualize the proliferative pattern along the long-axis of the brain under homeostasis (i.e. control) and at 1-dpl, 3-dpl, and 7-dpl. Note the global upregulation in EdU-labelling at 1-dpl compared with more localized cell proliferation at the injury site at 3-dpl. All brains are shown in dorsal view. White dashed circles denote the brain hemisphere in which the lesion was performed. Telencephalic lesions were performed as described previously[20]. dpl; days post lesion; OPT, optical projection tomography; OB, olfactory bulb; Tel, telencephalon; TeO, tectum opticum; Ce, cerebellum; LX, vagal lobe; SC, spinal cord.

Supplementary Figure 4. Macrophage response to tectal lesion. **(a)** Experimental design to investigate macrophage recruitment and proliferation at the site of lesion at 1-dpl and 3-dpl using the *Tg(mpeg1:mCherry)*^{g122} reporter line. **(b-d)** Distribution of resident microglia under homeostasis in the periventricular grey zone (PGZ) and superficial tectal layers (**b**) compared to increase in peripheral and local macrophages at 1-dpl (**c**) and 3-dpl (**d**) at the lesion canal. Red circles denote a change in macrophage morphology from an enlarged cell body with ramified processes under uninjured conditions (**b**) to a condensed, amoeboid-like state post-injury (**c-d**). Co-labelling with EdU demonstrates an increase in EdU-labelled cells post-injury (**b** vs. **c-d**) near the lesion canal, and the presence of EdU^{+}/mCherry^{+} proliferating macrophages at 1-dpl and 3-dpl in the superficial layers (**c-d**, white boxes). White boxes in **b-d** are shown at higher magnification in **e-g**. **(e-g)** EdU-negative macrophages with elongate morphology are seen in the superficial layers of control animals **(e**; red arrows**)**, whereas EdU^{+}/mCherry^{+} labelling is observed in a subpopulation of activated macrophages at 1-dpl **(f)** and 3-dpl **(g)** after injury (red arrows). **(h-i)** Co-labelling of L-plastin and Proliferating Cell Nuclear Antigen (PCNA) showing the absence of microglial proliferation in the unlesioned hemisphere **(h)** and L-plastin^{+}/PCNA^{+} subpopulations in the PGZ and superficial layers in the lesioned hemisphere (**i**; red arrows) at 3-dpl. In **c-d, i**, orange asterisk depicts the site of lesion canal. In all panels DAPI nuclear counterstaining (blue) was performed. In all cross-sectional images dorsal is oriented up. dpl, days post lesion.

Supplementary Figure 5. Nuclear β-catenin staining in qRG of stem cell zone 1 under homeostasis and injury. **(a-d)** Control (uninjured) tissue showing nuclear β-catenin expression in qRG of the quiescent RG layer (qRG-L) and sparse labelling in the upper neuronal layer (Neu-L). **(e-h)** Tissue following lesion at 3-dpl illustrating similar nuclear β-catenin staining as under control conditions in the qRG-L. High magnification insets in **a-h** depict the cell identified by the white arrow in each panel in single- and multi-channel images. **(i)** Quantification of the population size of gfap:GFP^{+}/βcat^{+} cells in the qRG-L between control and lesioned conditions (unpaired t-test, two-tailed: p = 0.3248). In panel **a-h** DAPI nuclear counterstaining (blue) was performed. In all cross-sectional images dorsal is oriented up. dpl, days post lesion.

Supplementary Figure 1

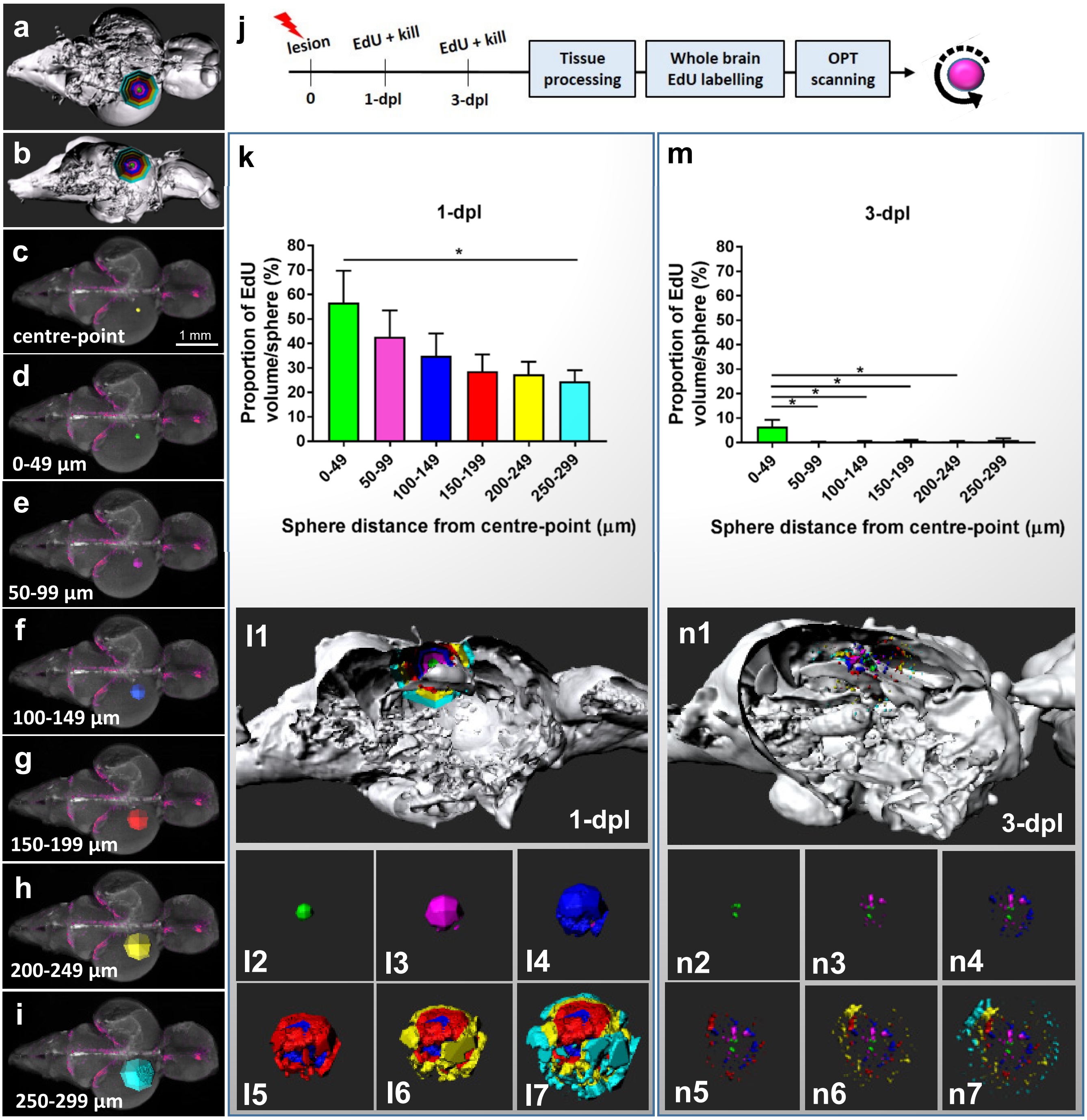

Supplementary Figure 2

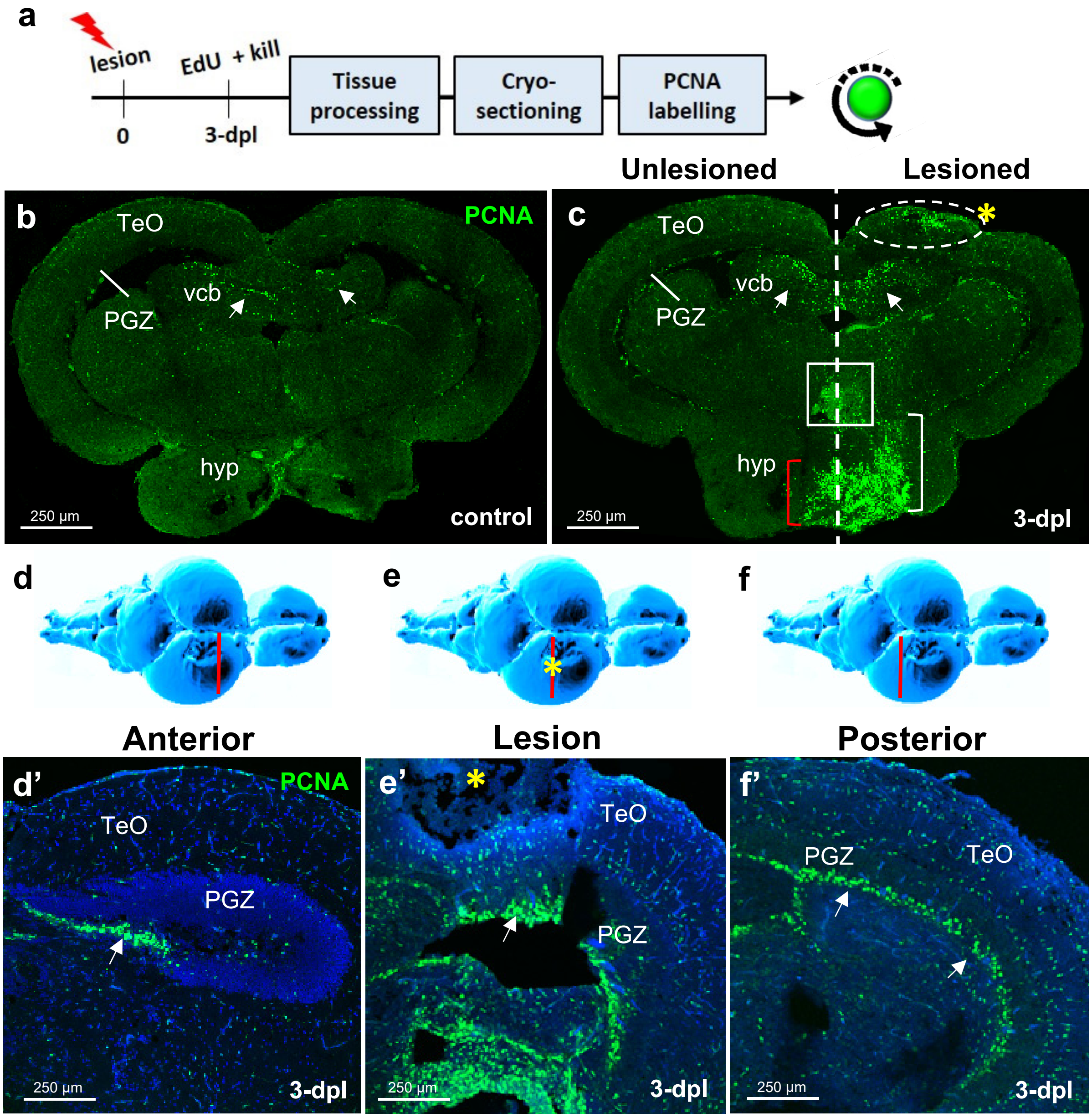

Supplementary Figure 3

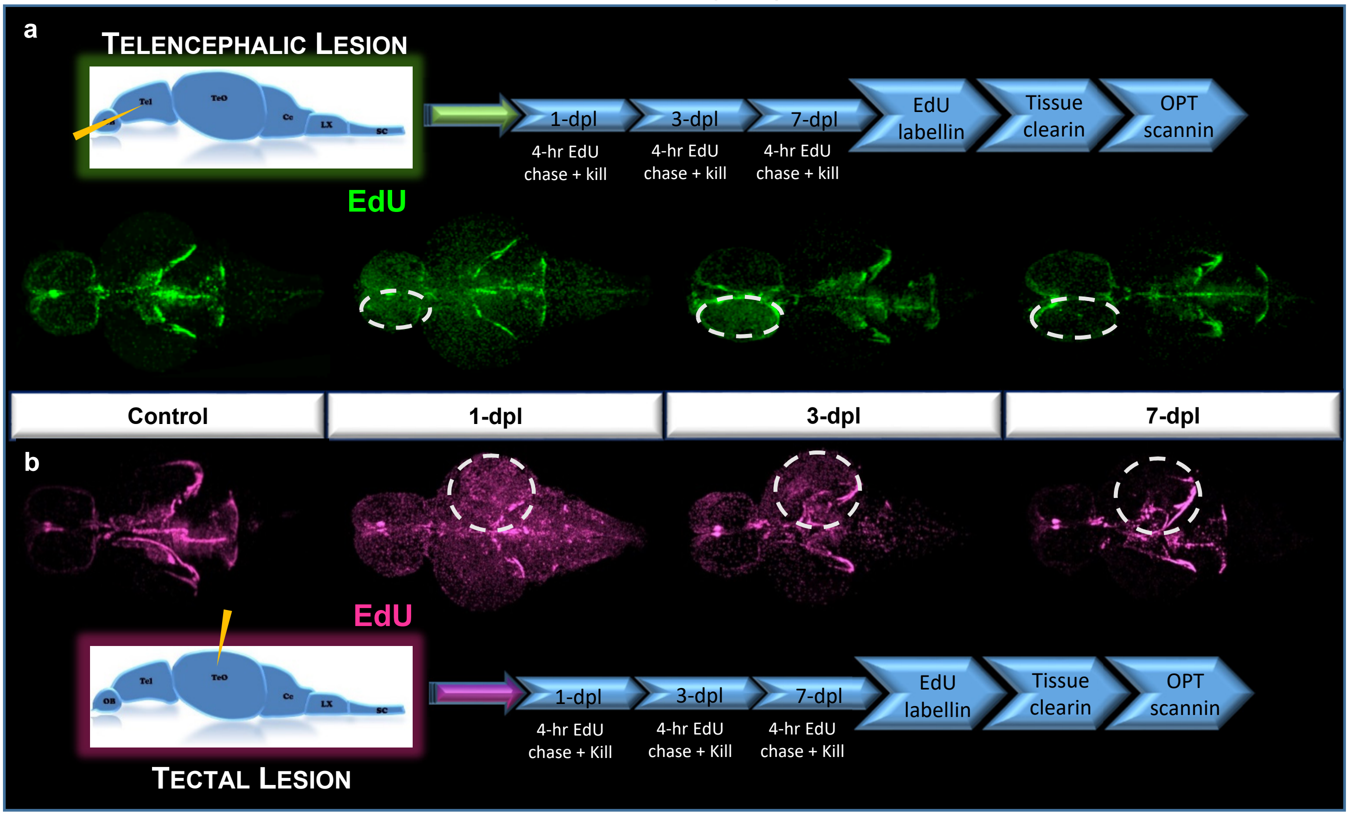

Supplementary Figure 4

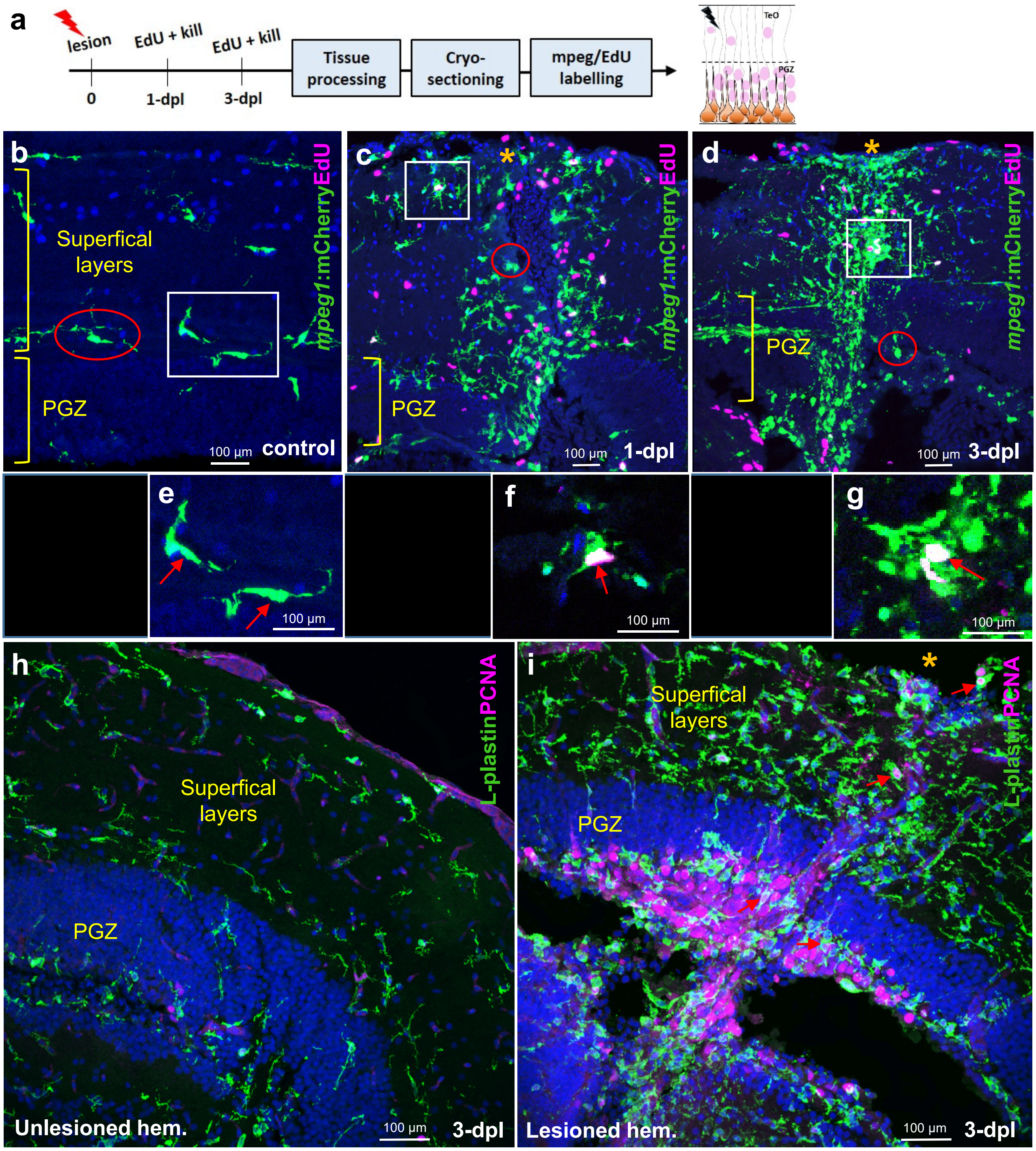

Supplementary Figure 5

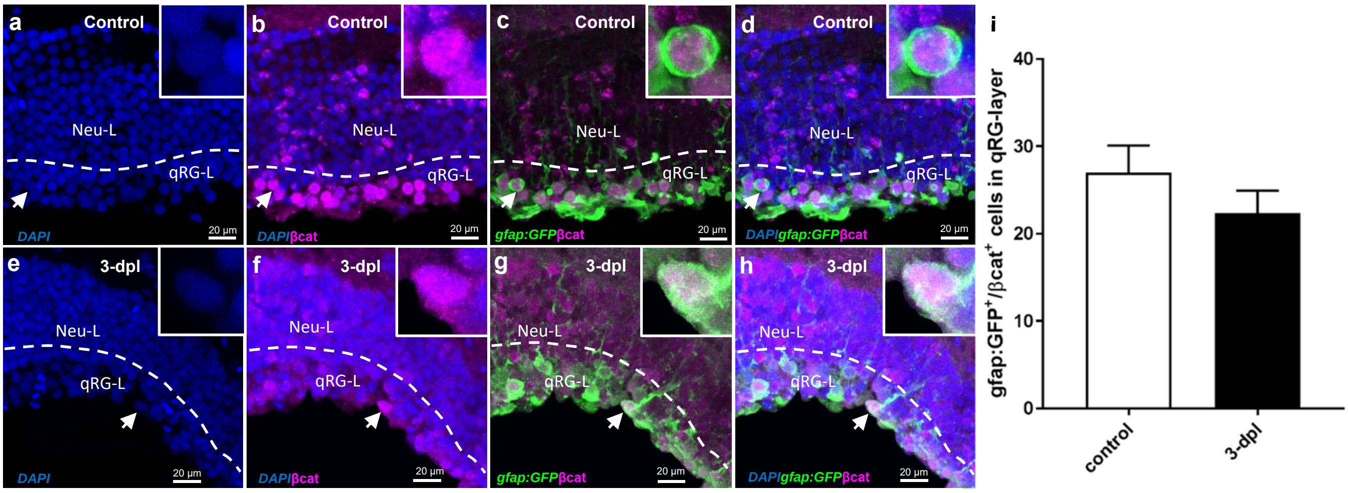

Supplement: Supplementary file 1 — Supplementary Figures 1–5 [file 41598_2019_40734_MOESM1_ESM.pdf]
